# Supplementary material for: Help-seeking behaviour and attitudes towards internet-administered psychological support among adolescent and young adults previously treated for cancer during childhood: protocol for a survey and embedded qualitative interview study in Sweden
Source: BMJ Open. 2021 Apr 8;11(4):e041350. doi: 10.1136/bmjopen-2020-041350 (PMC8039225; doi:10.1136/bmjopen-2020-041350)
Supplement: Author's manuscript [file bmjopen-2020-041350.draft_revisions.pdf]

BMJ Open

BMJ Open is committed to open peer review. As part of this commitment we make the peer review history of every article we publish publicly available.

When an article is published we post the peer reviewers' comments and the authors' responses online. We also post the versions of the paper that were used during peer review. These are the versions that the peer review comments apply to.

The versions of the paper that follow are the versions that were submitted during the peer review process. They are not the versions of record or the final published versions. They should not be cited or distributed as the published version of this manuscript.

BMJ Open is an open access journal and the full, final, typeset and author-corrected version of record of the manuscript is available on our site with no access controls, subscription charges or pay-per-view fees (http://bmjopen.bmj.com).

If you have any questions on BMJ Open's open peer review process please email info.bmjopen@bmj.com

BMJ Open

Help-seeking behaviour and attitudes towards internet-administered psychological support among adolescent and young adults previously treated for cancer during childhood: Protocol for a survey and embedded qualitative interview study in Sweden

Journal:	*BMJ Open*
Manuscript ID	bmjopen-2020-041350
Article Type:	Protocol
Date Submitted by the Author:	05-Jun-2020
Complete List of Authors:	Woodford, Joanne; Uppsala University, Clinical Psychology in Healthcare, Department of Women´s and Children´s Health<br>Månberg, Jenny; Uppsala University, Clinical Psychology in Healthcare, Department of Women´s and Children´s Health; Region Vasternorrland, Child and Adolescent Psychiatry<br>Cajander, Åsa; Uppsala University, Department of Information Technology<br>Enebrink, Pia; Karolinska Institute, Department of Clinical Neuroscience<br>Harila-Saari , Arja; Uppsala University, Pediatric Oncology, Department of Women's and Children's Health,<br>Hagström, Josefin; Uppsala University, Clinical Psychology in Healthcare, Department of Women's and Children's Health<br>Karlsson, Mathilda; Uppsala University, Clinical Psychology in Healthcare, Department of Women´s and Children´s Health<br>Placid Solimena, Hanna; Uppsala University, International Maternal and Child Health Care, Department of Women's and Children's Health<br>von Essen, Louise; Uppsala University, Clinical Psychology in Healthcare, Department of Women’s and Children’s Health
Keywords:	MENTAL HEALTH, Paediatric oncology < ONCOLOGY, PRIMARY CARE, Child & adolescent psychiatry < PSYCHIATRY, QUALITATIVE RESEARCH

SCHOLARONE™
Manuscripts

I, the Submitting Author has the right to grant and does grant on behalf of all authors of the Work (as defined in the below author licence), an exclusive licence and/or a non-exclusive licence for contributions from authors who are: i) UK Crown employees; ii) where BMJ has agreed a CC-BY licence shall apply, and/or iii) in accordance with the terms applicable for US Federal Government officers or employees acting as part of their official duties; on a worldwide, perpetual, irrevocable, royalty-free basis to BMJ Publishing Group Ltd ("BMJ") its licensees and where the relevant Journal is co-owned by BMJ to the co-owners of the Journal, to publish the Work in this journal and any other BMJ products and to exploit all rights, as set out in our licence.

The Submitting Author accepts and understands that any supply made under these terms is made by BMJ to the Submitting Author unless you are acting as an employee on behalf of your employer or a postgraduate student of an affiliated institution which is paying any applicable article publishing charge ("APC") for Open Access articles. Where the Submitting Author wishes to make the Work available on an Open Access basis (and intends to pay the relevant APC), the terms of reuse of such Open Access shall be governed by a Creative Commons licence – details of these licences and which Creative Commons licence will apply to this Work are set out in our licence referred to above.

Other than as permitted in any relevant BMJ Author's Self Archiving Policies, I confirm this Work has not been accepted for publication elsewhere, is not being considered for publication elsewhere and does not duplicate material already published. I confirm all authors consent to publication of this Work and authorise the granting of this licence.

For peer review only

Help-seeking behaviour and attitudes towards internet-administered psychological support among adolescent and young adults previously treated for cancer during childhood: Protocol for a survey and embedded qualitative interview study in Sweden

Joanne WOODFORD*[1] (joanne.woodford@kbh.uu.se), Jenny MÅNBERG[1,2] (jenny.manberg@rvn.se), Åsa CAJANDER[3] (asa.cajander@it.uu.se), Pia ENEBRINK[4] (pia.enebrink@ki.se), Arja HARILA-SAARI[5] (arja.harila-saari@kbh.uu.se), Josefin HAGSTRÖM[1] (josefin.hagstrom@kbh.uu.se), Mathilda KARLSSON[1] (mathilda.karlsson@kbh.uu.se), Hanna PLACID SOLIMENA[6] (hannaplacid@gmail.com), & Louise VON ESSEN[1] (louise-von.essen@kbh.uu.se).

Corresponding author

Dr Joanne Woodford, Clinical Psychology in Healthcare, Department of Women's and Children's Health. Uppsala University Dag Hammarskjölds väg 14B, 751 05 Uppsala, Sweden. E-mail: joanne.woodford@kbh.uu.se Telephone: 018-471-65 73

Affiliation

[1]Clinical Psychology in Healthcare, Department of Women´s and Children´s Health, Uppsala University, Uppsala, Sweden

[2]Child and Adolescent Psychiatry, Region Västernorrland, Sweden

[3]Visual Information and Interaction, Department of Information Technology, Uppsala University, Uppsala, Sweden

[4]Department of Clinical Neuroscience, Division of Psychology, Karolinska Institute, Stockholm, Sweden

[5]Pediatric Neurology / Pediatric Oncology, Department of Women's and Children's Health, Uppsala University, Uppsala, Sweden

[6]International Maternal and Child Health Care, Department of Women's and Children's Health, Uppsala University, Uppsala, Sweden

Word count: 4177

For peer review only

ABSTRACT

Introduction: A subgroup of adolescent and young adult childhood cancer survivors (AYACCS) are at an increased risk of psychological distress. Despite this, AYACCS experience difficulties accessing psychological support. E-mental health (e-MH) may offer a solution to reduce this treatment gap. However research examining e-MH for AYACCS has experienced difficulties with recruitment, retention, and adherence. Such difficulties may relate to: (1) help-seeking behaviour and/or (2) e-MH acceptability. Study objectives are to: (1) examine potential associations between health service use factors, informed by Andersen´s behavioural model of health services use, and help-seeking behaviour; (2) examine attitudes towards e-MH interventions; and (3) explore perceived need for mental health support; preferences for support; and barriers and facilitators to help-seeking.

Methods and analysis: An online and paper-based cross-sectional self-report survey (98 items) and embedded qualitative interview study across Sweden. Participants are 16-39 years, diagnosed with cancer when 0-18 years, and have completed successful cancer treatment. The survey examines: sociodemographic and clinical characteristics, actual help-seeking behaviour, attitudes towards e-MH, stigma of mental illness, mental health literacy, social support, and current symptoms of depression, anxiety, and stress. Survey respondents with past and/or current experience of mental health difficulties are invited into the qualitative interview study to explore: (1) perceived need for mental health support; (2) preferences for support; and (3) barriers and facilitators to help-seeking. Potential associations between health service use factors and help-seeking behaviour are examined using bivariate analyses and multivariable multinomial logistic regressions. Qualitative interviews are analysed using content analysis.

Discussion: Results will provide a greater understanding of factors associated with help-seeking behaviour, perceived need and preferences for mental health support, and attitudes towards e-MH in the population.

Ethics and dissemination: Ethical approval will be obtained from the Swedish Ethical Review Authority (Dnr: 2020/xxx). Results will be disseminated in scientific publications and academic conference presentations.

For peer review only

STRENGTHS AND LIMITATIONS OF THIS STUDY

- The mixed methods design may aid a rich and insightful understanding of the investigated phenomena.
- Results may be used to inform the development of acceptable, relevant, and accessible mental health interventions for adolescent and young adult childhood cancer survivors.
- Reasons for not receiving a mental health intervention are not explored and only those who report receiving support in the past six months are defined as help-seeking. This approach may exclude adolescent and young adult childhood cancer survivors who have attempted to seek formal help (help-seeking) but did not receive help.
- No conclusions concerning cause and effect can be drawn given the cross-sectional design

INTRODUCTION

Medical improvements have resulted in an overall five-year survival rate of 81.2 % from cancer diagnosed during childhood (0-18 years) in Northern Europe.[1] However, significant challenges, such as late effects, remain after treatment completion.[2, 3] A common late effect is symptoms of psychological distress, for example, posttraumatic stress, anxiety, and depression, symptoms of which have been found to persist decades post diagnosis.[4-6] Difficulties with psychological distress are further complicated given that adolescence is a period of increased vulnerability to mental health problems [7] with difficulties such as depression and anxiety commonly first appearing before 24 years of age.[8] Indeed, individuals diagnosed with cancer during childhood, adolescence, and young adulthood have been found to be at an increased likelihood of being prescribed hypnotics and anxiolytics [9, 10] of using antidepressant medication over the long-term,[11] and are at an elevated risk of suicide.[12] As such, adolescent and young adult childhood cancer survivors (AYACCS), commonly defined as childhood cancer survivors aged between 15-39 years [13] are particularly vulnerable to experiencing mental health difficulties. Despite a clear need for the provision of mental health support, a mental health treatment gap remains, with few AYACCS being offered professional psychological support.[14-17] Reasons for this treatment gap may pertain to a lack of qualified healthcare professionals, poor service provision, geographical barriers,[18] and public stigma (e.g., the general public holding negative beliefs about cancer survivors, for example, being less competent).[19]

E-mental health (e-MH), defined as the provision of psychological support via information technology and new media (e.g., online, smartphone applications, videoconferencing),[20] may represent a solution to reduce the mental health treatment gap.[21] One example of e-MH is internet-administered cognitive behavioural therapy (ICBT). ICBT has been found to be effective for a range of common mental health difficulties, such as depression and anxiety,[22] including for young adult populations [23] and has been found to be as effective as traditional face-to-face CBT.[24] E-MH interventions may help overcome practical barriers associated with accessing traditional face-to-face psychological support (e.g., geographical, financial, and time-related barriers) [25] that have been observed within the AYACCS population.[26] Further, e-MH is associated with anonymity and privacy [27] thus potentially overcoming barriers associated with public stigma. Indeed, research has identified that the provision of e-MH interventions may be a potential solution for the AYACCS population.[28-32] However, research examining e-MH support for an AYACCS population has encountered significant

For peer review only

challenges with recruitment, attrition, and internet use,[29, 33] challenges common in the field of e-MH research.[34-36] As such, there may be difficulties related to: (1) help-seeking behaviour in the population, and/or (2) the acceptability of e-MH.

Help-seeking behaviour

Low rates of formal help-seeking behaviour for mental health difficulties is common amongst an adolescent and young adult population in general.[37] A number of potential barriers to help-seeking have been identified, including stigma, embarrassment, and poor mental health literacy.[38, 39] Indeed, AYACCS report difficulties with stigma and alienation in relation to the cancer disease [40] and as such may be reluctant to identify themselves as experiencing mental health difficulties. Further, whilst social support has been found to facilitate help-seeking behaviour for mental health difficulties in adolescent and young adult populations,[38] AYACCS commonly report difficulties with social withdrawal and loneliness.[41, 42] However, at present there is a lack of research concerning help-seeking behaviour for mental health difficulties experienced by an AYACCS population.

A frequently used approach for identifying factors associated with help-seeking is Andersen´s behavioural model of health services use behavior.[43] The model hypothesizes a number of factors to be associated with help-seeking behaviour: (1) predisposing factors including sociodemographic variables (e.g., age and gender) and health related beliefs and knowledge;[44] (2) enabling factors relating to logistical elements to receiving care and resources (e.g., financial factors and social support);[45] (3) external environmental factors (e.g., location); and (4) need-related factors (e.g., symptoms of psychological distress). Recently the model has been adopted to examine factors associated with accessing both traditional and e-MH services within a young adult (18-39) population.[28] Interestingly, the study identified a weaker utilisation of e-MH by young people who had experienced negative childhood experiences during childhood (e.g., family conflict, bullying, or lack of affection) [28]. This finding is of particular importance seeing that negative experiences of childhood are normally associated with increased mental healthcare utilization.[46] Further, given evidence to suggest some AYACCS may experience bullying related to their cancer experience [47] and poor family functioning for example conflict [48] it may be important to examine whether negative childhood experiences are associated with reduced mental healthcare utilisation in an AYACCS population. Further, to the best of our knowledge, Andersen´s behavioural model of health services use has not yet been applied to an AYACCS population and factors associated with help-seeking in the population remain unclear.

Acceptability

Whilst adolescents appear to consider e-MH interventions to be potentially acceptable they still report a preference for face-to-face over e-MH interventions.[49] Indeed, there is some indication e-MH resources are not widely accessed by young people.[50] One potential difficulty may be related to what has been coined the '*technology push*', whereby e-MH solutions are designed to fit available technology, rather than to meet the unmet needs and preferences of end-users.[51] Further, research indicates poor uptake and ongoing use of e-MH interventions that have been implemented into healthcare settings within the general population.[52] As such, there is a need to understand preferences and attitudes towards e-MH interventions within specific populations,

For peer review only

via both quantitative and qualitative research, to inform future e-MH developments.[53] To the best of our knowledge, there has been no exploration to date of the attitudes and preferences towards e-MH interventions within an AYACCS population.

Objectives

Objectives are to: (1) examine potential association between health service use factors, informed by Andersen´s behavioural model of health services use (predisposing, enabling, environmental, and needs related), and help-seeking behaviour (actual help-seeking and type of help-seeking) within an AYACCS population; (2) to examine attitudes towards e-MH interventions, and (3) to explore: (1) perceived need for mental health support; (b) preferences for support; and (c) barriers and facilitators to help-seeking.

METHOD AND ANALYSIS

Study design

An online and paper-based, cross-sectional, self-report survey and embedded qualitative interview study across Sweden.

Eligibility criteria

Eligible participants will: (1) be 16-39 years at study start; (2) have been diagnosed with cancer when 0-18 years; (3) have completed successful cancer treatment (including relapses) at a minimum 3 months ago according to self-report; (4) be able to read and write in Swedish; and (5) currently reside in Sweden.

Recruitment

A multifaceted approach to recruitment will be adopted, using two main strategies:

Swedish Childhood Cancer Registry

Personal identification numbers of AYACCS will be obtained from the Swedish Childhood Cancer Registry (National Quality Registry, initiated in 1982). Next, addresses will be obtained via NAVET, a population registry held by the Swedish Tax Agency. NAVET is continuously updated with important information about the Swedish population, including deaths and can, as such, provide current addresses and information concerning the vital status of AYACCS. The Swedish Tax Agency will provide this information via a secure online file transfer system (e-transport) on the same day information is requested by the study team. Study invitation letters will be sent to AYACCS on the same day that information is provided via e-transport in order to minimise the likelihood of inviting a deceased AYACCS. AYACCS will be invited to participate by the study team on a weekly basis, using blocks of 100 until the target sample size has been reached. Blocks will be selected randomly, with minimisation used to ensure balance between factors that may affect outcomes: sex (male; female); current age (16-24; 25-39); and age at diagnosis (0-12; 13-18).

Study invitation packs sent to home addresses will include a study invitation letter, study information, a link to the online survey via the U-CARE-portal (www.u-care.se) a secure internet research platform to support data collection and provision of e-MH interventions [54] and a study log-in code. All potential participants identified

For peer review only

via the Swedish Childhood Cancer Registry and invited via mail-out will be provided with a link to the online survey on the U-CARE-portal and a study log-in code. In addition, given evidence to suggest the provision of choice between web-based and paper-based surveys can increase survey response rates amongst childhood cancer survivors,[55] We will include a paper-based survey and freepost envelope with each invitation letter.

Technology-based approaches

Considering that response rates to mail-out surveys have declined over the past two decades [56] we will also utilise technology-based recruitment approaches shown to be effective.[57] Indeed, social media recruitment is successful in recruiting young adult cancer survivor (aged 20–35 years) populations.[58] Specifically, we will identify community-based organisations and support groups with an online presence for AYACCS in Sweden. Examples may include: the Swedish Cancer Foundation and Childhood Cancer Foundation; and Young Cancer. Organisations and support groups will be asked to place advertisements on their websites; distribute advertisements via e-mail lists and e-newsletters; and post advertisements on social networking sites (e.g., Facebook, Twitter, and Instagram).

Each online advertisement will include brief study information, a link to the online survey on the U-CARE-portal, and a study log-in code unique to each organisation and technologically-based recruitment type to allow for the examination of success of each technology-based recruitment strategy. Organisations and support groups utilising social media will be encouraged to re-post the advertisement at regular intervals following evidence suggesting that re-posting is required to meet recruitment targets.[59]

Reasons for non-participation: AYACCS who do not wish to answer the survey will be able to actively opt out of the study and will be asked to complete a short questionnaire (provided in paper-format in study invitation packs and online via the U-CARE-portal) listing possible reasons for non-participation as informed by previous research.[60,61] It will be made clear that the provision of reasons for non-participation is optional.

Procedure

Online survey

Potential participants accessing the survey via the U-CARE-portal will be presented with study information in both text and video format. Those interested will be able to log into the U-CARE-portal using a study log-in code and will need to identify themselves via mobile telephone and/or Bank ID (a citizen authentication system). Potential participants will then be able to provide informed consent online and will be presented with brief screening questions asking for: (1) date of birth; (2) age when diagnosed with cancer; (3) date of last cancer treatment completed ; (4) able to read and write in Swedish (yes/no); and (5) whether currently residing in Sweden (yes/no). Eligible participants will be provided access to the online survey (see Outcomes). Upon completion of the survey a purposive sample of participants with past and/or present experience of mental health difficulties will be invited to take part in an interview (see Embedded qualitative interview study for further details). These participants will be provided with study information and online consent form, as well as asked to provide contact details.

For peer review only

Paper-based survey

Potential participants receiving study invitation via mail-out will be able to complete a paper-based survey. The survey includes a written consent form and brief screening questions as the online survey. Participants can return the paper-based survey to the study team using the freepost envelope provided in the study invitation pack. In addition, participants will be asked to provide consent if they agree to be contacted about participation in an interview study.

Reminders

Reminder study invitation packs will be resent at 2 weeks and 6 weeks after sending the initial study invitation to AYACCS who do not opt out of the study. It will be clearly stated in the study information sheet, that reminder letters will be sent to non-responders, with AYACCS provided with four methods to opt out of the study: (1) completing an online form via the Portal; (2) returning an opt out slip using a freepost envelope; (3) telephoning the research team; or (4) emailing the research team.

Survey items

An online and paper-based survey, consisting of 98 items in Swedish. The survey comprises eight subsections: (1) sociodemographic characteristics (11 items); (2) clinical cancer related characteristics (3 items); (3) actual help-seeking behaviour (2 items); (4) attitudes towards internet-administered interventions (17 items); (5) stigma of mental illness (5 items); (6) mental health literacy (26 items); (7) social support (12 items) and; (8) current symptoms of depression, anxiety, and stress (21 items). One open question at the end of the survey offers and opportunity provide any further information concerning emotional distress and preferences for support after childhood cancer. Potential predisposing, enabling, environmental, and need-related factors examined with the survey are summarised in Table 1.

Sociodemographic characteristics

Data on the following background and sociodemographic characteristics will be collected: (1) sex; (2) relationship status; (3) having children or not; (4) Swedish county of residence; (5) living in urban or rural area; (6) level of education; (7) employment status; (8) place of birth; (9) mother's place of birth; (10) father's place of birth; and (11) childhood negative events (bullying, family conflict, lack of affection).

Clinical cancer related characteristics

The following clinical characteristics related to cancer will be collected: (1) type of cancer (leukaemia, central nervous system tumour, solid tumour, lymphoma or other malignancy); (2) number of relapses (one or more than one); and (3) current late effects of cancer (multiple choice listed).

Actual help-seeking behaviour

The following items will be used to examine actual help-seeking behaviour: (1) emotional health difficulties associated with the cancer experienced. (2) the type of support received by those who accessed support in the past 6 months, and/or in the past (not including the past 6 months): (a) face-to-face individual or group support from mental health professional ; (b) face-to-face support from a general practitioner/family doctor; (c)

For peer review only

technology-administered mental health support (e.g., online therapy programme, smartphone application, video or teleconferencing); (d) technology-administered support from a general practitioner/family doctor (e.g., online, video, or teleconferencing); (e) social media support group; (f) phone help-line; (g) family, partner, or friend/s; (h) religious leader; and (i) other. Type of support will be categorized into: (1) face-to-face professional support; (2) technology-administered professional support; and (3) informal support (social media support group, family, partner, friends, and religious leader). Those who sought support for mental health difficulties in the past 6 months and received support from a health professional either face-to-face or technology assisted will be defined as 'help-seeking'.[62]

Attitudes towards internet-administered interventions

The 17-item e-Therapy Attitudes Measure [ETAM: 63, 64] will be adopted to measure attitudes towards internet-administered interventions, however, for the purpose of the present certain one item concerning health insurance companies was excluded due to not being relevant in the Swedish context. The questionnaire begins with definitions of three types of internet-administered interventions: (1) unguided internet-administered self-help programs; (2) therapist guided internet-administered self-help programs; and (3) psychological therapy delivered via videoconferencing. Subsequently, respondents are required to state which type of internet-administered intervention they would prefer, or state if they would not use internet-administered treatment at all. Subsequently, the ETAM is presented and explores attitudes towards internet-administered interventions. Specifically, respondents are instructed to state whether they agree with each statement on a 5-point rating scale ranging from 0 ("strongly disagree") to 4 ("strongly agree") and are asked to rate items considering therapist guided internet-administered self-help programs. The ETAM has been demonstrated to have good internal-consistency reliability ($\alpha = 0.89$).[64]

Symptoms of depression, anxiety, and stress

The Swedish version [65] of the (7-item depression, 7-item anxiety, and 7-item stress subscales) Depression Anxiety and Stress Scale [DASS-21: 66, 67] will be adopted to examine symptoms of depression, anxiety, and stress. The DASS-21 has adequate psychometric properties [68] and satisfactory test-retest reliability,[69] with support for the factor structure and convergent validity of the Swedish version.[65]

Stigma of mental illness

The 5-item Stigma Scale for Receiving Psychological Help [SSRPH: 70] will be adopted to measure public stigma, i.e. negative perceptions concerning the receipt of mental health services. The measure has demonstrated to be reliable and valid in studies with young adults [71] and adolescents.[72] Respondents rate degrees of agreement using a four-point Likert scale ranging from 0 ("strongly disagree") to 3 ("strongly agree"), with a higher score representing a higher level of stigma.

Social support

The 12-item Interpersonal Support Evaluation List [ISEL-12: 73] will be adopted to examine perceptions of social support. Responses are provided on a four-point Likert scale, ranging from 1 ("definitely false") to 4 ("definitely true").

For peer review only

Mental health literacy (MHL)

A 26-item multicomponent MHL measure, demonstrated to have good internal consistency (of 0.83 of the KR-20 coefficient = 0.83),[74] and will be adopted to assess knowledge-oriented, beliefs-oriented, and resource-oriented MHL. The first 22 items examining knowledge-orientated and beliefs-orientated MHL are answered on a five-point Likert scale ranging from 0 ("strongly disagree") to 4 ("strongly agree") and the option of "I don't know." The response format for the final 4 items, examining resource-orientated MHL, is "yes" or "no".

Table 1: Factors to be examined as per Andersen´s behavioural model of health services use

**Andersen´s behavioural model of health services use**	**Factors***
Predisposing	Sex (male/female/other)<br>Age (16-24/25-39)<br>Place of birth (born in Sweden/born outside of Sweden)<br>Childhood negative events (yes/no)<br>Level of education (≤upper secondary school/> upper secondary school)<br>Public stigma (SSRPH)<br>Mental health literacy (MHL)
Enabling	Type of cancer (leukemia/CNS tumor/solid tumor/lymphoma/other)<br>Relationship status<br>Employment status (yes/no/studying)<br>Social support (ISEL-12)
Need-related	Symptoms of depression (DASS-21 depression subscale)<br>Symptoms of anxiety (DASS-21 anxiety subscale)<br>Symptoms of stress (DASS-21 stress subscale)<br>Self-reported experience of mental health difficulties in past 6 months (yes/no)
Environmental	Region of Sweden (South/Mid/North)<br>Rural or urban area
Actual help-seeking behaviour (outcome)	Receipt of mental health support in the past 6 months (yes)<br>Type of mental health support received in the past 6 months (formal/technology/informal)

Footnote: *Outcomes selected informed by previous research [28, 37, 45, 62]

Translation of English language outcome measurements into Swedish

Currently, there are no Swedish versions of the following outcome measurements: (1) ETAM, (3) SSRPH, (4) ISEL, and (5) MLH. The following steps will be taken to translate: (1) one clinical psychologist, native Swedish speaker with advanced knowledge of English, will translate each outcome measurement to Swedish; and (2)

For peer review only

translations will be reviewed, discussed, and revised with a research assistant in our study team who is a native Swedish speaker with advanced knowledge of English. Professional back translation to English and discussion with the original author is out of scope for the present study due to time and resource limitations. However, permission to translate outcome measurements to Swedish will be sought.

Embedded qualitative interview study

An embedded qualitative interview study will be conducted to explore: (1) perceived need for mental health support; (2) preferences for support; and (3) barriers and facilitators to help-seeking. As this study will be examining mental health help-seeking, only respondents meeting the following criteria will be invited into the study: (1) self-reported current and/or past experience of mental health difficulties during life time (yes); and/or (2) DASS-21 depression score ≥5 and/or (3) DASS-21 anxiety score ≥4 and/or DASS-21 stress score ≥8. Respondents will be provided with full study information and an online consent form. Respondents meeting these criteria will be invited consecutively until data saturation is met.[75, 76] For those who send the consent form and reply slip via the post, those meeting the inclusion criteria will be contacted by the study team to provide full study information and obtain informed consent. Interviews will be conducted either via the telephone or a secure video-conferencing system on the U-CARE-portal. Interviews will be audio recorded with informed consent and transcribed with personal information removed. Participants will be informed about the recording and give approval when giving consent to the interview. An interview guide will be developed, consisting of open-ended questions, structured around the third research objective covering the following topics: (1) perceived need for mental health support; (2) preferences for support; and (3) barriers and facilitators to help-seeking behaviour. The interview guide will be partially informed by emerging survey results and findings from studies examining help-seeking behaviour in young adults.[28, 37, 45, 62] Interviews are estimated to last 45 to 90 minutes.

Data analysis

Statistical analysis

All statistical analyses will be conducted using SPSS V.26.0 software (IBM SPSS Statistics for Windows, V.26.0, 2019, Armonk, NY: IBM Corp.). Sociodemographic and clinical characteristics will be presented with descriptive statistics. The potential association between predicting factors as informed by Andersen´s behavioural model of health services use (predisposing, enabling, environmental, and need-related factors) and the dependent variable help-seeking behaviour will be examined using univariable and multivariable logistic regressions.[62] The multivariable logistic regression will be performed using the backward selection procedure. For both the univariable and multivariable logistic regressions, Odds Ratios will be presented, alongside 95% confidence intervals, and p-values.

Sample size estimation

The Swedish Childhood Cancer Registry has some 9,880 Swedish persons diagnosed when 0-18 years registered since 1982. However, some will have been classified as having a benign tumour, some have been registered twice, and some will be deceased. As such, the number of potentially eligible cases will be lower and estimated to be approximately 5,745. Sample size calculation indicated that a minimum of 365 participants

For peer review only

would be required, with a power of 0.9 and p<.05 (two-tailed). Assuming a response rate of approximately 40%, as indicated by a systematic review of participation rates in self-administered questionnaire studies with childhood cancer survivors [77] we estimate inviting 913 potential participants to achieve the required sample size.

Qualitative analysis

Audio recordings will be transcribed verbatim. NVivo V.12 software (NVivo qualitative data analysis software, V.12, 2018; QSR International Pty.) will be used to assist data analysis. A content analysis approach [78] will be adopted to analyse transcriptions. To ensure trustworthiness [79] the following strategies will be adopted: (1) two members of the research team, one researcher with experience of qualitative research and one research assistant trained in qualitative research, will analyse each interview separately with any discrepancies in analysis discussed; (2) discussion of emerging themes with the wider study team; (3) audit trails; (4) triangulation; and (5) disconfirming case analysis.[80] Further, extracts of data supporting each theme and subtheme will be presented to further improve the transparency of the analysis.[81]

Patient and public involvement

The protocol was developed without patient and public involvement (PPI). However, we will seek to involve AYACCS in data interpretation of the content analysis of the embedded semi-structured interviews. Specifically, a consultation approach to PPI will be adopted [82] with results of the content analysis presented to a panel of AYACCS research partners to explore whether themes identified by the research team reflect their own experiences. The perspective of the AYACCS research partners will be incorporated into the interpretation of results and their involvement will be reported in accordance with Guidance for Reporting Involvement of Patients and the Public (GRIPP) – short form.[83]

DISCUSSION

To the best of our knowledge, this study will be the first study to examine mental health help-seeking behaviour and attitudes towards e-MH support in an AYACCS population. Results will provide an understanding of: (1) potential health service use factors and help-seeking behaviour; (2) attitudes towards e-MH; and (3) perceived need and preferences for mental health support, alongside barriers and facilitators to help seeking in the population. Identification of factors associated with help-seeking behaviour may help identify potential targets to improve help-seeking behaviour in the future. For example, interventions could be developed to target factors investigated in this study and found to be negatively associated with help-seeking.[84] Further, increasing our understanding of attitudes towards e-MH support and preferences regarding the provision of mental health support in the population may inform the development of acceptable and relevant interventions for this underserved population.

ETHICS AND DISSEMINATION

The study will be approved by the Swedish Ethical Review Authority (Dnr: XXXX/XXX). The rights and welfare of participants will be ensured by all research being conducted in accordance with the Helsinki Declaration. Informed consent will be collected, ensuring participants are aware of requirements for study

For peer review only

participation. Participants will be free to withdraw from the study at any time, without providing reason. Contact details for the principal investigator (coauthor LvE), the U-CARE Health and Safety Officer, and Uppsala University Data Protection Officer will be provided to all participants should there be any cause for concern regarding the conduct of the study.

All data will be handled according to the Patient Data Act (2008:355) and General Data Protection Regulation (EU 2016/679). Data collected via the online survey via the U-CARE-portal will be securely stored on Uppsala University servers. Data collected via the paper-based survey will be entered into an Access Database, stored on Uppsala University servers. Paper questionnaires will be stored in locked secure filing cabinets, only accessible by authorised members of the study team. Participant contact details for the interview study will be stored separately on a USB stick in a locked secure filing cabinet, separate from study data. Interviews will be audio recorded, with audio files uploaded onto and stored on Uppsala University secure server, using participant identifier numbers and immediately deleted from devices. Interview transcripts will omit any personally identifiable data and will be stored on a secure server.

Survey results will be reported in line with the Checklist for Reporting Results of Internet e-Surveys (CHERRIES) [85] and results of the embedded qualitative interview study will be reported in accordance with the Standards for Reporting Qualitative Research (SRQR).[86] Results will be published in scientific publications in peer reviewed journals and conference presentations. Lay language summaries will also be provided to all community-based organisations and support groups who support recruitment into the study.

AUTHOR'S CONTRIBUTIONS

JW: Conceptualisation, methodology, writing - original draft, supervision, project administration, funding acquisition; JM: Methodology, resources, writing - original draft, project administration; ÅC: Writing – review and editing; PE: Writing – review and editing; AH-S: Writing – review and editing; JH: Resources, writing – review and editing, project administration; MK: Methodology, resources, writing – review and editing, project administration; HR: Resources, writing – review and editing, project administration; LvE: Methodology, Writing – review and editing, supervision, funding acquisition.

FUNDING

This work is supported by U-CARE, which is a Strategic Research environment funded by the Swedish Research Council (dnr 2009–1093) and Fredrik O Ingrid Thurings Stiftelse (grant number 2018-00437).

COMPETING INTERESTS

The authors have no competing interests to declare.

COPYRIGHT AND AUTHORS' RIGHTS

I Joanne Woodford, the Submitting Author has the right to grant and does grant on behalf of all authors of the Work (as defined in the below author licence), an exclusive licence and/or a non-exclusive licence for contributions from authors who are: i) UK Crown employees; ii) where BMJ has agreed a CC BY licence shall

For peer review only

apply, and/or iii) in accordance with the terms applicable for US Federal Government officers or employees acting as part of their official duties; on a worldwide, perpetual, irrevocable, royalty-free basis to BMJ Publishing Group Ltd ("BMJ") its licensees and where the relevant Journal is co-owned by BMJ to the co-owners of the Journal, to publish the Work in [BMJ Open] and any other BMJ products and to exploit all rights, as set out in our licence.

For peer review only

References

1. Gatta G, Botta L, Rossi S, et al. Childhood cancer survival in Europe 1999-2007: results of EUROCARE-5--a population-based study. *Lancet Oncol* 2014;15:35-47. doi:10.1016/S1470-2045(13)70548-5 [published Online First: 5 December 2013].
2. Armstrong GT, Kawashima T, Leisenring W, et al. Aging and risk of severe, disabling, life-threatening, and fatal events in the childhood cancer survivor study. *J Clin Oncol* 2014;32:1218-27. doi:10.1200/JCO.2013.51.1055 [published Online First: 17 March 2014].
3. Bhakta N, Liu Q, Ness KK, et al. The cumulative burden of surviving childhood cancer: an initial report from the St. Jude lifetime cohort study. *Lancet* 2017;390:2569–82. doi:10.1016/S0140-6736(17)31610-0
4. Ander M, Grönqvist H, Cernvall M, et al. Development of health-related quality of life and symptoms of anxiety and depression among persons diagnosed with cancer during adolescence: a 10-year follow-up study. *Psychooncology* 2016;25:582-89. doi:26361085 [published Online First: 10 September 2015].
5. Brinkman TM, Zhu L, Zeltzer LK, et al. Longitudinal patterns of psychological distress in adult survivors of childhood cancer. *Br J Cancer* 2013;109:1373-81. doi:10.1038/bjc.2013.428 [published Online First: 23 July 2013].
6. Seitz DC, Besier T, Debatin KM, et al. Posttraumatic stress, depression and anxiety among adult long-term survivors of cancer in adolescence. *Eur J Cancer* 2010;46:1596-06. doi:10.1016/j.ejca.2010.03.001[published Online First: 8 April 2010].
7. Patton GC, Sawyer SM, Santelli JS, et al. Our future: a lancet commission on adolescent health and wellbeing. *Lancet* 2016;387:2423-78. doi:10.1016/S0140-6736(16)00579-1 [published Online First: 9 May 2016].
8. Blackmore SJ. Adolescence and mental health. *Lancet* 2019;393:2030-31. doi:10.1016/S0140-6736(19)31013-X [published Online First: 16 May 2019].
9. Ahomäki R, Kero A, Koivisto M, et al. Purchases of antidepressants after cancer at a young age in Finland. *Int J Cancer* 2019:144:1227-33. doi:10.1002/ijc.31942 25 [published Online First: 25 October 2018].
10. Johannsdottir IM, Loge JH, Kiserud CE, et al. Increased prescription rates of anxiolytics and hypnotics to survivors of cancer in childhood, adolescence, and young adulthood-a population-based study. *Pediatr Blood Cancer* 2018;65:e26860. doi:10.1002/pbc.26860 [published Online First: 27 October 2017].
11. Brinkman TM, Ullrich NJ, Zhang N, et al. Prevalence and predictors of prescription psychoactive medication use in adult survivors of childhood cancer: a report from the childhood cancer survivor study. *J Cancer Surviv* 2013;7:104–14. doi:10.1007/s11764-012-0250-x [published Online First: 8 December 2012].
12. Gunnes MW, Lie RT, Bjørge T, et al. Suicide and violent deaths in survivors of cancer in childhood, adolescence and young adulthood-a national cohort study. *Int J Cancer* 2017;140:575-80. doi: 10.1002/ijc.30474 [published Online First: 17 October 2016].
13. Bleyer A, Barr R, Ries L, etal. *Cancer in adolescents and young adults.* Springer International Publishing, 2017.
14. Bibby H, White V, Thompson K, et al. What are the unmet needs and care experiences of adolescents and young adults with cancer? A systematic review. *J Adolesc Young Adult Oncol* 2017;6:6-30. doi:10.1089/jayao.2016.0012 [published Online First: 1 May 2017].

For peer review only

15. Kaul S, Avila JC, Mutambudzi M, et al. Mental distress and health care use among survivors of adolescent and young adult cancer: a cross-sectional analysis of the national health interview survey. *Cancer* 2017;123:869-78. doi:10.1002/cncr.30417 [published Online First: 17 November 2016].

16. Olsson M, Jarfelt M, Pergert P, et al. Experiences of teenagers and young adults treated for cancer in Sweden. *Eur J Pncol Nurs* 2015;19:575-81. doi:10.1016/j.ejon.2015.03.003[published Online First: 13 April 2015].

17. Svedberg P, Einberg EL, Wärnestål P, et al. Support from healthcare services during transition to adulthood – experiences of young adult survivors of pediatric cancer. *Eur J Oncol Nursing* 2016;21:105-12. doi:10.1016/j.ejon.2016.02.008 [published Online First: 23 February 2016].

18. Rebello TJ, Marques A, Gureje O, et al. Innovative strategies for closing the mental health treatment gap globally. *Curr Opin Psychiatry* 2014;27:308-14. doi:10.1097/YCO.0000000000000068 [published Online First: July 2014].

19. Kim MA, Yi J. Life after cancer: how does public stigma increase psychological distress of childhood cancer survivors? *Int J Nurs Stud* 2014;51:1605-14. doi:10.1016/j.ijnurstu.2014.04.005 [published Online First: 5 May 2014].

20. Riper H, Andersson G, Christensen H, et al. Theme issue on e-mental health: a growing field in internet research. *J Med Internet Res* 2010;12:e74. doi:10.2196/jmir.1713 [published Online First: 19 December 2010].

21. Patel V, Saxena S, Lund C, et al. The lancet commission on global mental health and sustainable development. *Lancet* 2018;392:1553-98. doi:10.1016/S0140-6736(18)31612-X [published Online First: 9 October 2018].

22. Andersson G. Internet interventions: past, present and future. *Internet Interv* 2018;12:181- 88. doi: 10.1016/j.invent.2018.03.008 [published Online First: 6 April 2018].

23. Bunge EL, Dickter B, Jones MK, et al. Behavioural intervention technologies and psychotherapy with youth: a review of the literature. *Curr Psychiatry Rev* 2016;12:14–28. doi:10.2174/1573400511666150930232254

24. Carlbring P, Andersson G, Cuijpers P, et al. Internet-based vs. face-to-face cognitive behaviour therapy for psychiatric and somatic disorders: an updated systematic review and meta-analysis. *Cogn Behav Ther* 2018;47:1-18. doi:10.1080/16506073.2017.1401115 [published Online First: 7 December 2017].

25. Sansom-Daly UM, Wakefield CE, Bryant RA, et al. Online group-based cognitive-behavioural therapy for adolescents and young adults after cancer treatment: a multicenter randomised controlled trial of recapture life-AYA. *BMC Cancer* 2012;12. doi:10.1186/1471-2407-12-339 [published Online First: 3 August 2012].

26. Hagström, J, Ander, M, Cernvall, et al. Heeding the psychological concerns of young cancer survivors: a single-arm feasibility trial of CBT and a cognitive behavioural conceptualization of distress. *PeerJ* 2020;8:e8714. doi:10.7717/peerj.8714 [published Online First: 19 March 2020].

27. Younes N, Chollet A, Menard E, et al. E-mental health care among young adults and help-seeking behaviours: a transversal study in a community sample. *J Med Internet Res* 2015;17:e123. doi: 10.2196/jmir.4254 [published Online First: 15 May 2015].

28. Ander M, Wikman A, Ljótsson B, et al. Guided internet-administered self-help to reduce symptoms of anxiety and depression among adolescents and young adults diagnosed with cancer during adolescence (U-

For peer review only

CARE: YoungCan): a study protocol for a feasibility trial. BMJ Open 2017;7: e013906. doi:10.1136/bmjopen-2016-013906 [published Online First: 27 January 2017].

29. Ander M. *Cancer during adolescence - psychological consequences and development of psychological treatment.* 2017.

30. Barnett M, McDonnell G, DeRosa A, et al. Psychosocial outcomes and interventions among cancer survivors diagnosed during adolescence and young adulthood (AYA): a systematic review. *J Cancer Surviv* 2016;10:814-31. doi:10.1007/s11764-016-0527-6 [published Online First: 26 February 2016].

31. Devine KA, Viola AS, Coups EJ, et al. Digital health interventions for adolescent and young adult cancer survivors. *JCO Clin Cancer Inform* 2018;2:1-15. doi:10.1200/CCI.17.00138 [published Online First: 29 June 2018].

32. Moody L, Turner A, Osmond J, et al. Web-based self-management for young cancer survivors: consideration of user requirements and barriers to implementation. *J Cancer Surviv* 2015;9:188–200. doi:10.1007/s11764-014-0400-4 [published Online First: 19 September 2014].

33. Seitz DC, Knaevelsrud C, Duran G, et al. Efficacy of an internet-based cognitive-behavioral intervention for long-term survivors of pediatric cancer: a pilot study. *Support Care Cancer* 2014;22:2075–83. doi:10.1007/s00520-014-2193-4 [published Online First: 18 March 2014].

34. van Ballegooijen W, Cuijpers P, van Straten A, et al. Adherence to internet-based and face-to-face cognitive behavioural therapy for depression: a meta-analysis. *PLoS One* 2014;9:e100674. doi:10.1371/journal.pone.0100674 [published Online First: 16 July 2014].

35. Nilsson A, Sörman K, Klingvall J, et al. MyCompass in a Swedish context - lessons learned from the transfer of a self-guided intervention targeting mental health problems. *BMC Psychiatry* 2019;19:51. doi:10.1186/s12888-019-2039-1[published Online First: 31 January 2019].

36. Woodford J, Farrand P, Bessant M, et al. Recruitment into a guided internet based CBT (iCBT) intervention for depression: lesson learnt from the failure of a prevalence recruitment strategy. *Contemp Clin Trials* 2011;32:641-48. doi:10.1016/j.cct.2011.04.013 [published Online First: 5 May 2011].

37. Rapp AM, Chavira DA, Sugar CA, et al. Integrated primary medical-behavioural health care for adolescent and young adult depression: predictors of service use in the youth partners in care trial. *J Pediatr Psychol* 2017;42:1051-64. doi:10.1093/jpepsy/jsx057 [published Online First: 25 March 2017].

38. Gulliver A, Griffiths KM, Christensen H. Perceived barriers and facilitators to mental health help-seeking in young people: a systematic review. *BMC Psychiatry* 2010;10. doi:10.1186/1471-244X-10-113 [published Online First: 30 December 2010].

39. Kaess M, Bauer S. Promoting help-seeking using e-technology for adolescents: the ProHEAD consortium. *Trials* 2019;20:72. doi:10.1186/s13063-018-3162-x [published Online First: 22 January 2019].

40. Lehmann V, Grönqvist H, Engvall G, et al. Negative and positive consequences of adolescent cancer 10 years after diagnosis: an interview-based longitudinal study in Sweden. *Psychooncology* 2014;23:1229-35. doi:10.1002/pon.3549 [published Online First: 15 April 2014].

41. Ander M, Thorsell Cederberg J, von Essen L, et al. Exploration of psychological distress experienced by survivors of adolescent cancer reporting a need for psychological support. *PLoS One* 2018;13:e0195899. doi:10.1371/journal.pone.0195899

For peer review only - http://bmjopen.bmj.com/site/about/guidelines.xhtml

42. Wilford J, Buchbinder D, Fortier MA, et al. "She was a little social butterfly": a qualitative analysis of parent perception of social functioning in adolescent and young adult brain tumor survivors. *J Pediatr Oncol Nurs* 2017;34:239-49. doi:10.1177/1043454216688660 [published Online First: 1 February 2017].

43. Andersen RM. Revisiting the behavioral model and access to medical care: does it matter? *J Health Soc Behav* 1995;36:1-10. doi:10.2307/2137284

44. Bock JO, Hajek A, König HH. The longitudinal association between psychological factors and health care use. *Health Serv Res* 2018;53:1065-91. doi:10.1111/1475-6773.12679 [published Online First: 15 March 2017].

45. Gudleski GD, Satchidanand N, Dunlap LJ, et al. Predictors of medical and mental health care use in patients with irritable bowel syndrome in the United States. *Behav Res Ther* 2017;88:65-75. doi:10.1016/j.brat.2016.07.006 [published Online First: 19 January 2017].

46. Mills V, Van HM, Baur J, et al. Predictors of mental health service utilisation in a non-treatment seeking epidemiological sample of Australian adults. *Community Ment Health J* 2012;48:511-21. doi:10.1007/s10597-011-9439-0 [published Online First: 31 October 2011].

47. Mooney-Somers J, Lewis P, Kerridge I. Discursive constructions of youth cancer: findings from creative methods research with healthy young people. *J Cancer Surviv* 2016;10:427-36. doi:10.1007/s11764-015-0488-1 [published Online First: 12 November 2015].

48. van Schoors M, Caes L, Knoble NB, et al. Systematic review: associations between family functioning and child adjustment after pediatric cancer diagnosis: a meta-analysis. *J Pediatr Psychol* 2017;42:6-18. doi:10.1093/jpepsy/jsw070 [published Online First: 24 September 2016].

49. Sweeney GM, Donovan CL, March S, et al. Logging into therapy: adolescent perceptions of online therapies for mental health problems. *Internet interv* 2016;15:93-9. doi:10.1016/j.invent.2016.12.001 [published Online First: 24 December 2016].

50. Wetterlin FM, Mar MY, Neilson EK, et al. eMental health experiences and expectations: a survey of youths' web-based resource preferences in Canada. *J Med Internet Res* 2014;16:e29. doi:10.2196/jmir.3526 [published Online First: 17 December 2014].

51. Hollis C, Morris R, Martin J, et al. Technological innovations in mental healthcare: harnessing the digital revolution. *Br J Psychiatry* 2015;206:263-265. doi:10.1192/bjp.bp.113.142612 [published Online First: 2 January 2018].

52. Fleming T, Bavin L, Lucassen M, et al. Beyond the trial: systematic review of real-world uptake and engagement with digital self-help interventions for depression, low mood, or anxiety. *J Med Internet Res* 2018;20:e199. dio:10.2196/jmir.9275[published Online First: 6 June 2018].

53. Apolinário-Hagen J, Kemper J, Stürmer C. Public acceptability of e-mental health treatment services for psychological problems: a scoping review. *JMIR Ment Health* 2017;4:e10. doi:10.2196/mental.6186 [published Online First: 3 April 2017].

54. Grönqvist H, Olsson EMG, Johansson B, et al. Fifteen challenges in establishing a multidisciplinary research program on eHealth research in university settings: a case study. *J Med Internet Res* 2017;19:e173. doi:10.2196/jmir.7310 [published Online First: 23 May 2017].

55. Kilsdonk E, van Dilmen-den Broeder E, van der Pal HJ, et al. Effect of web-based versus paper-based questionnaires and follow-up strategies on participation rates of Dutch childhood cancer survivors: a

For peer review only

randomized controlled trial. *JMIR Cancer* 2015;1:e11. doi:10.2196/cancer.3905 [published Online First: 24 November 2015].

56. Brick JM, Williams D. Explaining rising nonresponse rates in cross-sectional surveys. *AAPSS* 2013;645:36–59. doi:10.1177/0002716212456834 [published Online First: 26 November 2012].
57. Batterham PJ. Recruitment of mental health survey participants using internet advertising: content, characteristics and cost effectiveness. *Int J Methods Psychiatr Res* 2014;23:184-91. doi: 10.1002/mpr.1421[published Online First: 24 February 2014].
58. Gorman JR, Roberts SC, Dominick, SA. A Diversified Recruitment Approach Incorporating Social Media Leads to Research Participation Among Young Adult-Aged Female Cancer Survivors. *J Adolesc Young Adult Oncol* 2014:3: 59–5. doi:10.1089/jayao.2013.0031 [published Online First: 1 June 2014].
59. O'Connor A, Jackson L, Goldsmith L, et al. Can I get a retweet please? Health research recruitment and the twittersphere. *J Adv Nurs* 2014;70:599-09. doi:10.1111/jan.12222 [published Online First: 4 Augusti 2013].
60. Barnes M, Wiles N, Morrison J, et al. Exploring patients' reasons for declining contact in a cognitive behavioral therapy randomized controlled trial in primary care. *Br J Gen Pract* 2012;62:e371–77. doi: 10.3399/bjgp12X641492 [published Online First: 1 May 2012].
61. Woodford J, Wikman A, Cernvall M, et al. Study protocol for a feasibility study of an internet-administered, guided, CBT-based, self-help intervention (ENGAGE) for parents of children previously treated for cancer. *BMJ Open* 2018;8:e023708. doi:10.1136/bmjopen-2018-023708 [published Online First: June 2018].
62. Boerema AM, Kleiboer A, Beekman ATet al. Determinants of help-seeking behavior in depression: a cross-sectional study. *BMC Psychiatry* 2016;16. doi:10.1186/s12888-016-0790-0 [published Online First: 23 March 2016].
63. Apolinário-Hagen J, Vehreschild V, Alkoudmani RM. Current views and perspectives on e-mental health: an exploratory survey study for understanding public attitudes toward internet-based psychotherapy in Germany. *JMIR Ment Health* 2017;4:e8. doi:10.2196/mental.6375 [published Online First: 23 February 2017].
64. Apolinário-Hagen J, Harrer M, Kählke F, et al. Public attitudes toward guided internet-based therapies: web-based survey study. *JMIR Ment Health* 2018;5:e10735. doi:10.2196/10735 [published Online First: 15 May 2018].
65. Alfonsson S, Wallin E, Maathz P. Factor structure and validity of the depression, anxiety and stress scale-21 in Swedish translation. *J Psychiatr Ment Health Nurs* 2017;24:154-62. doi:10.1111/jpm [published Online First: 25 January 2017].
66. Brown TA, Chorpita BF, Korotitsch W, et al. Psychometric properties of the depression anxiety stress scales (DASS) in clinical samples. *Behav Res Ther* 1997;35:79-89. doi:10.1016/s0005-7967(96)00068-x [published Online First: 15 June 1998].
67. Ng F, Trauer T, Dodd S, et al. The validity of the 21-item version of the depression anxiety stress scales as a routine clinical outcome measure. *Acta Neuropsychiatr* 2007;19:304-10. doi:10.1111/j.1601-5215.2007.00217.x [published Online First: 24 June 2014].

For peer review only

68. Sinclair SJ, Siefert CJ, Slavin-Mulford JM, et al. Psychometric evaluation and normative data for the depression, anxiety, and stress scales-21 (DASS-21) in a nonclinical sample of U.S. adults. *Eval Health Prof* 2012;35:259-79. doi:10.1177/0163278711424282 [published Online First: 18 October 2011].

69. Bottesi G, Ghisi M, Altoè G, etal. The Italian version of the depression anxiety stress scales-21: factor structure and psychometric properties on community and clinical samples. *Compr Psychiatry* 2015;60:170-81. doi: 10.1016/j.comppsych.2015.04.005 [published Online First: 15 April 2015].

70. Komiya N, Good GE, Sherrod NB. Emotional openness as a predictor of college students' attitudes toward seeking psychological help. *J Couns Psychol* 2000;47:138–43. doi:10.1037/AJ022-0167,47.1.138

71. Bathje GJ, Pryor JB. The relationships of public and self-stigma to seeking mental health services. *J Ment Health Couns* 2011;33:161–76. doi:10.17744/mehc.33.2.g632039274160411 [published Online First: 5 April 2019].

72. Pinto MD, Greenblatt AM, Hickman RL, et al. Assessing the critical parameters of eSMART-MH: a promising avatar-based digital therapeutic intervention to reduce depressive symptoms. *Perspect Psychiatr Care* 2016;52:157-68. doi:10.1111/ppc.12112 [published Online First: 19 March 2015].

73. Cohen S, Mermelstein R, Kamarck T, *et al*. Measuring the functional components of social support. In: Sarason IG, Sarason BR, eds. *Social support: theory, research, and applications*. Martinus Nijhoff 1985. 73–94.

74. Jung H, von Stenberg K, Davis K. Expanding a measure of mental health literacy: development and validation of multicomponent mental health literacy measure. *Psychiatry Res* 2016;243:278-86. doi:10.1016/j.addr.2016.07.002 [published Online First: 14 July 2016].

75. Fusch PI, Ness LR. Are we there yet? Data saturation in qualitative research. *Qual Rep* 2015;20:1408-1416.

76. Guest G, Bunce A, Johnson L. How many interviews are enough? An experiment with data saturation and variability. *Sage journal* 2006;18:59-82. doi:10.1177/1525822X05279903 [published Online First: 1 February 2006].

77. Kilsdonk E, Wendel E, van Dulmen-den Broeder E, et al. Participation rates of childhood cancer survivors to self-administered questionnaires: a systematic review. *Eur J Cancer Care* 2017;26:e12462. doi:10.1111/ecc.12462 [published Online First: 16 February 2016].

78. Elo S, Kyngäs H. The qualitative content analysis process. *J Adv Nurs*. 2008;62:107-15. doi:10.1111/j.1365-2648.2007.04569.x [published Online First: 18 March 2008].

79. Krefting L. Rigor in qualitative research: the assessment of trustworthiness. *Am J Occup Ther* 1991;45:214-22. doi:10.5014/ajot.45.3.214 [published Online First: 1 March 1991].

80. Patton MQ. Enhancing the quality and credibility of qualitative analysis. *Health Serv Res* 1999;34:1189–1208.

81. Elliott R, Fischer CT, Rennie DL. Evolving guidelines for publication of qualitative research studies in psychology and related fields. *Br J Clin Psychol*. 1999;38:215-29. doi:10.1348/014466599162782

82. Jennings H., Slade M., Bates P. et al. Best practice framework for patient and public involvement (PPI) in collaborative data analysis of qualitative mental health research: methodology development and refinement. *BMC Psychiatry*. 2018:18. doi:10.1186/s12888-018-1794-8 [published Online First: 28 June 2018].

For peer review only

83. Staniszewska S, Brett J, Simera I, et al. GRIPP2 reporting checklists: tools to improve reporting of patient and public involvement in research. *BMJ* 2017;358:j3453. doi:10.1136/bmj.j3453 [published Online First: 2 August 2017].

84. Kauer SD, Mangan C, Sanci L. Do online mental health services improve help-seeking for young people? A systematic review. *J Med Internet Res* 2014;16:e66. doi:10.2196/jmir.3103 [published Online First: 4 March 2014].

85. Eysenbach G. Improving the quality of web surveys: the checklist for reporting results of internet e-surveys (CHERRIES). J Med Internet Res 2004;6:e34. doi:10.2196%2Fjmir.6.3.e34 [published Online First: 29 September 2004].

86. O'Brien BC, Harris IB, Beckman TJ, et al. Standards for reporting qualitative research: a synthesis of recommendations. *Academic Medicine* 2014;89:1245-51. doi:10.1097/ACM.0000000000000388

For peer review only

BMJ Open

Help-seeking behaviour and attitudes towards internet-administered psychological support among adolescent and young adults previously treated for cancer during childhood: Protocol for a survey and embedded qualitative interview study in Sweden

Journal:	*BMJ Open*
Manuscript ID	bmjopen-2020-041350.R1
Article Type:	Protocol
Date Submitted by the Author:	01-Nov-2020
Complete List of Authors:	Woodford, Joanne; Uppsala University, Clinical Psychology in Healthcare, Department of Women´s and Children´s Health<br>Månberg, Jenny; Uppsala University, Clinical Psychology in Healthcare, Department of Women´s and Children´s Health; Region Vasternorrland, Child and Adolescent Psychiatry<br>Cajander, Åsa; Uppsala University, Department of Information Technology<br>Enebrink, Pia; Karolinska Institute, Department of Clinical Neuroscience<br>Harila-Saari , Arja; Uppsala University, Pediatric Oncology, Department of Women's and Children's Health,<br>Hagström, Josefin; Uppsala University, Clinical Psychology in Healthcare, Department of Women's and Children's Health<br>Karlsson, Mathilda; Uppsala University, Clinical Psychology in Healthcare, Department of Women´s and Children´s Health<br>Placid Solimena, Hanna; Uppsala University, International Maternal and Child Health Care, Department of Women's and Children's Health<br>von Essen, Louise; Uppsala University, Clinical Psychology in Healthcare, Department of Women’s and Children’s Health
**Primary Subject Heading**:	Mental health
Secondary Subject Heading:	Oncology
Keywords:	MENTAL HEALTH, Paediatric oncology < ONCOLOGY, PRIMARY CARE, Child & adolescent psychiatry < PSYCHIATRY, QUALITATIVE RESEARCH

SCHOLARONE™
Manuscripts

I, the Submitting Author has the right to grant and does grant on behalf of all authors of the Work (as defined in the below author licence), an exclusive licence and/or a non-exclusive licence for contributions from authors who are: i) UK Crown employees; ii) where BMJ has agreed a CC-BY licence shall apply, and/or iii) in accordance with the terms applicable for US Federal Government officers or employees acting as part of their official duties; on a worldwide, perpetual, irrevocable, royalty-free basis to BMJ Publishing Group Ltd ("BMJ") its licensees and where the relevant Journal is co-owned by BMJ to the co-owners of the Journal, to publish the Work in this journal and any other BMJ products and to exploit all rights, as set out in our licence.

The Submitting Author accepts and understands that any supply made under these terms is made by BMJ to the Submitting Author unless you are acting as an employee on behalf of your employer or a postgraduate student of an affiliated institution which is paying any applicable article publishing charge ("APC") for Open Access articles. Where the Submitting Author wishes to make the Work available on an Open Access basis (and intends to pay the relevant APC), the terms of reuse of such Open Access shall be governed by a Creative Commons licence – details of these licences and which Creative Commons licence will apply to this Work are set out in our licence referred to above.

Other than as permitted in any relevant BMJ Author's Self Archiving Policies, I confirm this Work has not been accepted for publication elsewhere, is not being considered for publication elsewhere and does not duplicate material already published. I confirm all authors consent to publication of this Work and authorise the granting of this licence.

For peer review only

Help-seeking behaviour and attitudes towards internet-administered psychological support among adolescent and young adults previously treated for cancer during childhood: Protocol for a survey and embedded qualitative interview study in Sweden

Joanne WOODFORD*[1] (joanne.woodford@kbh.uu.se), Jenny MÅNBERG[1,2] (jenny.manberg@rvn.se), Åsa CAJANDER[3] (asa.cajander@it.uu.se), Pia ENEBRINK[4] (pia.enebrink@ki.se), Arja HARILA-SAARI[5] (arja.harila-saari@kbh.uu.se), Josefin HAGSTRÖM[1] (josefin.hagstrom@kbh.uu.se), Mathilda KARLSSON[1] (mathilda.karlsson@kbh.uu.se), Hanna PLACID SOLIMENA[6] (hannaplacid@gmail.com), & Louise VON ESSEN[1] (louise-von.essen@kbh.uu.se).

Corresponding author

Dr Joanne Woodford, Clinical Psychology in Healthcare, Department of Women's and Children's Health. Uppsala University Dag Hammarskjölds väg 14B, 751 05 Uppsala, Sweden. E-mail: joanne.woodford@kbh.uu.se Telephone: 018-471-65 73

Affiliation

[1]Clinical Psychology in Healthcare, Department of Women´s and Children´s Health, Uppsala University, Uppsala, Sweden

[2]Child and Adolescent Psychiatry, Region Västernorrland, Sweden

[3]Visual Information and Interaction, Department of Information Technology, Uppsala University, Uppsala, Sweden

[4]Department of Clinical Neuroscience, Division of Psychology, Karolinska Institute, Stockholm, Sweden

[5]Pediatric Neurology / Pediatric Oncology, Department of Women's and Children's Health, Uppsala University, Uppsala, Sweden

[6]International Maternal and Child Health Care, Department of Women's and Children's Health, Uppsala University, Uppsala, Sweden

Word count: 5115

For peer review only

ABSTRACT

Introduction: A subgroup of adolescent and young adult childhood cancer survivors (AYACCS) are at an increased risk of psychological distress. Despite this, AYACCS experience difficulties accessing psychological support. E-mental health (e-MH) may offer a solution to reduce this treatment gap. However research examining e-MH for AYACCS has experienced difficulties with recruitment, retention, and adherence. Such difficulties may relate to: (1) help-seeking behaviour and/or (2) e-MH acceptability. The overall aims of the study are to: (1) examine potential associations between health service use factors, informed by Andersen´s behavioural model of health services use, and help-seeking behaviour; (2) examine attitudes towards e-MH interventions; and (3) explore perceived need for mental health support; past experience of receiving mental health support; preferences for support; and barriers and facilitators to help-seeking.

Methods and analysis: An online and paper-based cross-sectional self-report survey (98 items) and embedded qualitative interview study across Sweden, with a target sample size of n=365. Participants are 16-39 years, diagnosed with cancer when 0-18 years, and have completed successful cancer treatment. The survey examines: sociodemographic and clinical characteristics, actual help-seeking behaviour, attitudes towards e-MH, stigma of mental illness, mental health literacy, social support, and current symptoms of depression, anxiety, and stress. Survey respondents with past and/or current experience of mental health difficulties are invited into the qualitative interview study to explore: (1) perceived need for mental health support; (2) past experience of receiving mental health support; (3) preferences for support; and (4) barriers and facilitators to help-seeking. Potential associations between health service use factors and help-seeking behaviour are examined using bivariate analyses and multivariable multinomial logistic regressions. Qualitative interviews are analysed using content analysis. **Ethics and dissemination:** Ethical approval will be obtained from the Swedish Ethical Review Authority (Dnr: 2020/xxx). Results will be disseminated in scientific publications and academic conference presentations.

For peer review only

STRENGTHS AND LIMITATIONS OF THIS STUDY

- The mixed methods design may aid a rich and insightful understanding of the investigated phenomena.
- Results may be used to inform the development of acceptable, relevant, and accessible mental health interventions for adolescent and young adult childhood cancer survivors.
- Reasons for not receiving a mental health intervention are not explored and only those who report receiving support in the past six months are defined as help-seeking. This approach may exclude adolescent and young adult childhood cancer survivors who have attempted to seek formal help (help-seeking) but did not receive help.
- No conclusions concerning cause and effect can be drawn given the cross-sectional design

INTRODUCTION

Medical improvements have resulted in an overall five-year survival rate of 81.2 % from cancer diagnosed during childhood (0-18 years) in Northern Europe.[1] However, significant challenges, such as late effects, remain after treatment completion.[2, 3] Survivors of childhood cancer report a number of long-term adverse physical and developmental outcomes [4-6] and social and economic impacts, including poorer educational and occupational achievements.[7,8] A further common late effect is symptoms of psychological distress, for example, posttraumatic stress, anxiety, and depression, symptoms of which have been found to persist decades post diagnosis.[9-11] Difficulties with psychological distress are further complicated given that adolescence is a period of increased vulnerability to mental health problems [12] with difficulties such as depression and anxiety commonly first appearing before 24 years of age.[13] Indeed, individuals diagnosed with cancer during childhood, adolescence, and young adulthood have been found to be at an increased likelihood of being prescribed hypnotics and anxiolytics [14, 15] of using antidepressant medication over the long-term,[16] and are at an elevated risk of suicide.[17] As such, adolescent and young adult childhood cancer survivors (AYACCS), commonly defined as childhood cancer survivors aged between 15-39 years [18] are particularly vulnerable to experiencing adverse psychological and social outcomes, and there is a significant need for long-term care and support.[5] However, despite a clear need for the provision of mental health support, a mental health treatment gap remains, with few AYACCS being offered professional psychological support.[19-22] Reasons for this treatment gap may pertain to a lack of qualified healthcare professionals, poor service provision, geographical barriers,[23] and public stigma (e.g., the general public holding negative beliefs about cancer survivors, for example, being less competent).[24]

E-mental health (e-MH), defined as the provision of psychological support via information technology and new media (e.g., online, smartphone applications, videoconferencing),[25] may represent a solution to reduce the mental health treatment gap.[26] One example of e-MH is internet-administered cognitive behavioural therapy (ICBT). ICBT has been found to be effective for a range of common mental health difficulties, such as depression and anxiety,[27] including for young adult populations [28] and has been found to be as effective as traditional face-to-face CBT.[29] E-MH interventions may help overcome practical barriers associated with accessing traditional face-to-face psychological support (e.g., geographical, financial, and time-related barriers) [30] that have been observed within the AYACCS population.[31] Further, e-MH is associated with anonymity

For peer review only

and privacy [32] thus potentially overcoming barriers associated with public stigma. Indeed, research has identified that the provision of e-MH interventions may be a potential solution for the AYACCS population.[33-38] However, research examining e-MH support for an AYACCS population has encountered challenges with recruitment, attrition, and internet use.[34, 38] For example, one study examining the feasibility and acceptability of e-MH recruited only 28 participants over a 12 month period and experienced almost 30% attrition.[38] Another feasibility study of e-MH for adolescents and young adults who had experienced cancer during the adolescent period recruited only 6 participants from a total of 320 potential participants invited into the study, and experienced a 100% attrition rate.[34] Indeed, a recent review of e-Health interventions for AYACCS concluded that engaging the population was challenging, and there is currently a lack of literature concerning how to better engage the population.[36] However, challenges with recruitment, attrition, and internet use are common in the field of e-MH research.[39-41] Potentially, challenges with recruitment, attrition, and internet use may be related to help-seeking behaviour and the acceptability of e-MH for the population.

Help-seeking behaviour

Low rates of formal help-seeking behaviour for mental health difficulties is common amongst an adolescent and young adult population in general.[42] A number of potential barriers to help-seeking have been identified, including stigma, embarrassment, and poor mental health literacy.[43, 44] Indeed, AYACCS report difficulties with stigma and alienation in relation to the cancer disease [45] and as such may be reluctant to identify themselves as experiencing mental health difficulties. Further, whilst social support has been found to facilitate help-seeking behaviour for mental health difficulties in adolescent and young adult populations,[43] AYACCS commonly report difficulties with social withdrawal and loneliness.[46, 47] However, at present there is a lack of research concerning help-seeking behaviour for mental health difficulties experienced by an AYACCS population.

A frequently used approach for identifying factors associated with help-seeking is Andersen´s behavioural model of health services use behavior.[48] The model hypothesizes a number of factors to be associated with help-seeking behaviour: (1) predisposing factors including sociodemographic variables (e.g., age and gender) and health related beliefs and knowledge;[49] (2) enabling factors relating to logistical elements to receiving care and resources (e.g., financial factors and social support);[50] (3) external environmental factors (e.g., location); and (4) need-related factors (e.g., symptoms of psychological distress). Recently the model has been adopted to examine factors associated with accessing both traditional and e-MH services within a young adult (18-39) population.[32] Interestingly, the study identified a weaker utilisation of e-MH by young people who had experienced negative childhood experiences during childhood (e.g., family conflict, bullying, or lack of affection) [32]. This finding is of particular importance seeing that negative experiences of childhood are normally associated with increased mental healthcare utilization.[51] Further, given evidence to suggest some AYACCS may experience bullying related to their cancer experience [52] and poor family functioning for example conflict [53] it may be important to examine whether negative childhood experiences are associated with reduced mental healthcare utilisation in an AYACCS population. Further, to the best of our knowledge, Andersen´s behavioural model of health services use has not yet been applied to an AYACCS population and factors associated with help-seeking in the population remain unclear.

Acceptability

Whilst adolescents appear to consider e-MH interventions to be potentially acceptable they still report a preference for face-to-face over e-MH interventions.[54] Indeed, there is some indication e-MH resources are not widely accessed by young people.[55] One potential difficulty may be related to what has been coined the '*technology push*', whereby e-MH solutions are designed to fit available technology, rather than to meet the unmet needs and preferences of end-users.[56] Further, research indicates poor uptake and ongoing use of e-MH interventions that have been implemented into healthcare settings within the general population.[57] As such, there is a need to understand preferences and attitudes towards e-MH interventions within specific populations, via both quantitative and qualitative research, to inform future e-MH developments.[58] To the best of our knowledge, there has been no exploration to date of the attitudes and preferences towards e-MH interventions within an AYACCS population. In addition, tailoring e-MH interventions to specific patient groups has been associated with increased effectiveness [59] and adherence.[60] Indeed, rates of attrition may be reduced, and adherence increased, if the perspective of the population using the intervention is taken into account when developing e-MH interventions.[61] Further, research into the psychological needs of an AYACCS population indicates a need for increased focus on the specific psychological and psychosocial needs of the population to enable to provision of more tailored support to the cancer experience.[62] As such, there is need to explore AYCACCs general preferences for psychological support, including potential intervention content.

Study aims and research questions

The overall aims of the study are to: (1) examine potential associations between health service use factors, informed by Andersen´s behavioural model of health services use, and help-seeking behaviour; (2) examine attitudes towards e-MH interventions; and (3) explore perceived need and past experiences of mental health support; preferences for support; and barriers and facilitators to help-seeking. Specifically, the following research questions will be examined:

(1) Are there associations between predisposing, enabling, environmental, and needs related health service use factors, informed by Andersen´s behavioural model of health, and help-seeking behaviour (actual help-seeking and type of help-seeking) within an AYACCS population?

(2) What attitudes are held by an AYACCS population towards e-MH interventions?

(3) What is the perceived need for mental health support within an AYACCS population?

(4) What are AYACCS experiences of past receipt of cancer specific and non-cancer experience specific mental health support?

(5) What preferences do an AYACCS population hold towards mental health support related to their cancer experience?

(6) What are the barriers and facilitators do an AYACCA population experience seeking help for mental health support related to their cancer experience?

METHOD AND ANALYSIS

Study design

For peer review only

An online and paper-based, cross-sectional, self-report survey and embedded qualitative interview study across Sweden.

Eligibility criteria

Eligible participants will: (1) be 16-39 years at study start; (2) have been diagnosed with cancer when 0-18 years; (3) have completed successful cancer treatment (including relapses) at a minimum 3 months ago according to self-report; (4) be able to read and write in Swedish; and (5) currently reside in Sweden.

Recruitment

A multifaceted approach to recruitment will be adopted, using two main strategies:

Swedish Childhood Cancer Registry

Personal identification numbers of AYACCS will be obtained from the Swedish Childhood Cancer Registry (National Quality Registry, initiated in 1982). Next, addresses will be obtained via NAVET, a population registry held by the Swedish Tax Agency. NAVET is continuously updated with important information about the Swedish population, including deaths and can, as such, provide current addresses and information concerning the vital status of AYACCS. The Swedish Tax Agency will provide this information via a secure online file transfer system (e-transport) on the same day information is requested by the study team. Study invitation letters will be sent to AYACCS on the same day that information is provided via e-transport in order to minimise the likelihood of inviting a deceased AYACCS. AYACCS will be invited to participate by the study team on a weekly basis, using blocks of 100 until the target sample size has been reached. Blocks will be selected randomly, with minimisation used to ensure balance between factors that may affect outcomes: sex (male; female); current age (16-24; 25-39); and age at diagnosis (0-12; 13-18).

Study invitation packs sent to home addresses will include a study invitation letter, study information, a link to the online survey via the U-CARE-portal (www.u-care.se) a secure internet research platform to support data collection and provision of e-MH interventions [63] and a study log-in code. All potential participants identified via the Swedish Childhood Cancer Registry and invited via mail-out will be provided with a link to the online survey on the U-CARE-portal and a study log-in code. In addition, given evidence to suggest the provision of choice between web-based and paper-based surveys can increase survey response rates amongst childhood cancer survivors,[64] we will include a paper-based survey and freepost envelope with each invitation letter.

Technology-based approaches

Considering that response rates to mail-out surveys have declined over the past two decades [65] we will also utilise technology-based recruitment approaches shown to be effective.[66] Indeed, social media recruitment is successful in recruiting young adult cancer survivor (aged 20–35 years) populations.[67] Specifically, we will identify community-based organisations and support groups with an online presence for AYACCS in Sweden. Examples may include: the Swedish Cancer Foundation and Childhood Cancer Foundation; and Young Cancer. Organisations and support groups will be asked to place advertisements on their websites; distribute

For peer review only

advertisements via e-mail lists and e-newsletters; and post advertisements on social networking sites (e.g., Facebook, Twitter, and Instagram).

Each online advertisement will include brief study information, a link to the online survey on the U-CARE-portal, and a study log-in code unique to each organisation and technologically-based recruitment type to allow for the examination of success of each technology-based recruitment strategy. Organisations and support groups utilising social media will be encouraged to re-post the advertisement at regular intervals following evidence suggesting that re-posting is required to meet recruitment targets.[68]

Reasons for non-participation: AYACCS who do not wish to answer the survey will be able to actively opt out of the study and will be asked to complete a short questionnaire (provided in paper-format in study invitation packs and online via the U-CARE-portal) listing possible reasons for non-participation as informed by previous research.[69,70] It will be made clear that the provision of reasons for non-participation is optional.

Sample size estimation

The Swedish Childhood Cancer Registry has some 9,880 Swedish persons diagnosed when 0-18 years registered since 1982. However, some will have been classified as having a benign tumour, some have been registered twice, and some will be deceased. As such, the number of potentially eligible cases will be lower and estimated to be approximately 5,745. Sample size calculation indicated that a minimum of 365 participants would be required, with a power of 0.9 and $p<.05$ (two-tailed). Assuming a response rate of approximately 40%, as indicated by a systematic review of participation rates in self-administered questionnaire studies with childhood cancer survivors [71] we estimate inviting 913 potential participants to achieve the required sample size.

Procedure

Online survey

Potential participants accessing the survey via the U-CARE-portal will be presented with study information in both text and video format. Participants will not be offered any financial reimbursements for taking part in the survey. Those interested will be able to log into the U-CARE-portal using a study log-in code and will need to identify themselves via mobile telephone and/or Bank ID (a citizen authentication system). Potential participants will then be able to provide informed consent online and will be presented with brief screening questions asking for: (1) date of birth; (2) age when diagnosed with cancer; (3) date of last cancer treatment completed; (4) able to read and write in Swedish (yes/no); and (5) whether currently residing in Sweden (yes/no). Eligible participants will be provided access to the online survey (see Outcomes). The online survey will be open for a period of six weeks. Each item completed in the survey is auto-saved and participants are also able to select "save and continue later" if they wish to return to the survey before submitting. Upon completion of the survey a purposive sample of participants with past and/or present experience of mental health difficulties will be invited to take part in an interview (see Embedded qualitative interview study for further details). These participants will be provided with study information and online consent form, as well as asked to provide contact details.

For peer review only

Paper-based survey

Potential participants receiving study invitation via mail-out will be able to complete a paper-based survey. The survey includes a written consent form and brief screening questions as the online survey. Participants can return the paper-based survey to the study team using the freepost envelope provided in the study invitation pack. In addition, participants will be asked to provide consent if they agree to be contacted about participation in an interview study.

Reminders

Reminder study invitation packs will be resent at 2 weeks and 6 weeks after sending the initial study invitation to AYACCS who do not opt out of the study. It will be clearly stated in the study information sheet, that reminder letters will be sent to non-responders, with AYACCS provided with four methods to opt out of the study: (1) completing an online form via the Portal; (2) returning an opt out slip using a freepost envelope; (3) telephoning the research team; or (4) emailing the research team. In addition, participants who consent to complete the online survey will receive reminders via SMS and email to complete the survey at 4 weeks, 2 weeks, and 2 days prior to the survey closing.

Survey items

An online and paper-based survey, consisting of 98 items in Swedish. The survey comprises eight subsections: (1) sociodemographic characteristics (11 items); (2) clinical cancer related characteristics (3 items); (3) actual help-seeking behaviour (2 items); (4) attitudes towards internet-administered interventions (17 items); (5) stigma of mental illness (5 items); (6) mental health literacy (26 items); (7) social support (12 items) and; (8) current symptoms of depression, anxiety, and stress (21 items). One open question at the end of the survey offers and opportunity provide any further information concerning emotional distress and preferences for support after childhood cancer. Potential predisposing, enabling, environmental, and need-related factors examined with the survey are summarised in Table 1.

Sociodemographic characteristics

Data on the following background and sociodemographic characteristics will be collected: (1) sex; (2) relationship status; (3) having children or not; (4) Swedish county of residence; (5) living in urban or rural area; (6) level of education; (7) employment status; (8) place of birth; (9) mother's place of birth; (10) father's place of birth; and (11) childhood negative events (bullying, family conflict, lack of affection).

Clinical cancer related characteristics

The following clinical characteristics related to cancer will be collected: (1) type of cancer (leukaemia, central nervous system tumour, solid tumour, lymphoma or other malignancy); (2) number of relapses (zero, one or more than one); and (3) current late effects of cancer (multiple choice listed).

Actual help-seeking behaviour

For peer review only

The following items will be used to examine actual help-seeking behavior: (1) emotional health difficulties related to the childhood cancer experience; (2) the type of support received by those who accessed support in the past 6 months, and/or in the past (not including the past 6 months): (a) face-to-face individual or group support from mental health professional; (b) face-to-face support from a general practitioner/family doctor; (c) technology-administered mental health support (e.g., online therapy programme, smartphone application, video or teleconferencing); (d) technology-administered support from a general practitioner/family doctor (e.g., online, video, or teleconferencing); (e) social media support group; (f) phone help-line; (g) family, partner, or friend/s; (h) religious leader; and (i) other. Type of support will be categorized into: (1) face-to-face professional support; (2) technology-administered professional support; and (3) informal support (social media support group, family, partner, friends, and religious leader). Those who sought support for mental health difficulties in the past 6 months and received support from a health professional either face-to-face or technology assisted will be defined as 'help-seeking'.[72]

Attitudes towards internet-administered interventions

The 17-item e-Therapy Attitudes Measure [ETAM: 73, 74] will be adopted to measure attitudes towards internet-administered interventions, however, for the purpose of the present certain one item concerning health insurance companies was excluded due to not being relevant in the Swedish context. The questionnaire begins with definitions of three types of internet-administered interventions: (1) unguided internet-administered self-help programs; (2) therapist guided internet-administered self-help programs; and (3) psychological therapy delivered via videoconferencing. Subsequently, respondents are required to state which type of internet-administered intervention they would prefer, or state if they would not use internet-administered treatment at all. Subsequently, the ETAM is presented and explores attitudes towards internet-administered interventions. Specifically, respondents are instructed to state whether they agree with each statement on a 5-point rating scale ranging from 0 ("strongly disagree") to 4 ("strongly agree") and are asked to rate items considering therapist guided internet-administered self-help programs. The ETAM has been demonstrated to have good internal-consistency reliability ($\alpha = 0.89$).[74]

Symptoms of depression, anxiety, and stress

The Swedish version [75] of the (7-item depression, 7-item anxiety, and 7-item stress subscales) Depression Anxiety and Stress Scale [DASS-21: 76, 77] will be adopted to examine symptoms of depression, anxiety, and stress. The DASS-21 has adequate psychometric properties [78] and satisfactory test-retest reliability,[79] with support for the factor structure and convergent validity of the Swedish version.[75]

Stigma of mental illness

The 5-item Stigma Scale for Receiving Psychological Help [SSRPH: 80] will be adopted to measure public stigma, i.e. negative perceptions concerning the receipt of mental health services. The measure has demonstrated to be reliable and valid in studies with young adults [81] and adolescents.[82] Respondents rate degrees of agreement using a four-point Likert scale ranging from 0 ("strongly disagree") to 3 ("strongly agree"), with a higher score representing a higher level of stigma.

For peer review only

Social support

The 12-item Interpersonal Support Evaluation List [ISEL-12: 83] will be adopted to examine perceptions of social support. Responses are provided on a four-point Likert scale, ranging from 1 (“definitely false”) to 4 (“definitely true”).

Mental health literacy (MHL)

A 26-item multicomponent MHL measure, demonstrated to have good internal consistency (of 0.83 of the KR-20 coefficient = 0.83),[84] and will be adopted to assess knowledge-oriented, beliefs-oriented, and resource-oriented MHL. The first 22 items examining knowledge-orientated and beliefs-orientated MHL are answered on a five-point Likert scale ranging from 0 (“strongly disagree”) to 4 (“strongly agree”) and the option of “I don’t know.” The response format for the final 4 items, examining resource-orientated MHL, is “yes” or “no”.

Table 1: Factors to be examined as per Andersen´s behavioural model of health services use

**Andersen´s behavioural model of health services use**	**Factors***
Predisposing	Sex (male/female/other)<br>Age (16-24/25-39)<br>Time since first cancer treatment (years)<br>Place of birth (born in Sweden/born outside of Sweden)<br>Childhood negative events (yes/no)<br>Level of education (≤upper secondary school/> upper secondary school)<br>Public stigma (SSRPH)<br>Mental health literacy (MHL)
Enabling	Type of cancer (leukemia/CNS tumor/solid tumor/lymphoma/other)<br>Relationship status<br>Employment status (yes/no/studying)<br>Social support (ISEL-12)
Need-related	Symptoms of depression (DASS-21 depression subscale)<br>Symptoms of anxiety (DASS-21 anxiety subscale)<br>Symptoms of stress (DASS-21 stress subscale)<br>Self-reported experience of mental health difficulties in past 6 months (yes/no)
Environmental	Region of Sweden (South/Mid/North)<br>Rural or urban area
Actual help-seeking behaviour (outcome)	Receipt of mental health support in the past 6 months (yes)<br>Type of mental health support received in the past 6 months (formal/technology/informal)

Footnote: *Outcomes selected informed by previous research [32, 42, 50, 72]

For peer review only

Translation of English language outcome measurements into Swedish

Currently, there are no Swedish versions of the following outcome measurements: (1) ETAM, (3) SSRPH, (4) ISEL, and (5) MLH. The following steps will be taken to translate: (1) one clinical psychologist, native Swedish speaker with advanced knowledge of English, will translate each outcome measurement to Swedish; and (2) translations will be reviewed, discussed, and revised with a research assistant in our study team who is a native Swedish speaker with advanced knowledge of English. Professional back translation to English and discussion with the original author is out of scope for the present study due to time and resource limitations. However, permission to translate outcome measurements to Swedish will be sought.

Embedded qualitative interview study

An embedded qualitative interview study will be conducted to explore: (1) perceived need for mental health support; (2) past experience of receiving mental health support; (3) preferences for support; and (4) barriers and facilitators to help-seeking. As this study will be examining mental health help-seeking, only respondents meeting the following criteria will be invited into the study: (1) self-reported current and/or past experience of mental health difficulties during life time (yes); and/or (2) DASS-21 depression score ≥5 and/or (3) DASS-21 anxiety score ≥4 and/or DASS-21 stress score ≥8. Respondents will be provided with full study information and an online consent form. Respondents meeting these criteria will be invited consecutively until thematic data saturation is met.[85, 86] As the decision concerning whether data saturation has been met is made during the analytic process it is difficult to determine the number of interviews a priori, however we may anticipate interviewing approximately 20-30 participants.[87] For participants who send the consent form and reply slip via the post, those meeting the inclusion criteria will be contacted by the study team to provide full study information and obtain informed consent. Interviews will be conducted either via the telephone or a secure video-conferencing system on the U-CARE-portal. Interviews will be audio recorded with informed consent and transcribed with personal information removed. Participants will be informed about the recording and give approval when giving consent to the interview. An interview guide will be developed, consisting of open-ended questions, structured around the third research objective covering the following topics: (1) perceived need for mental health support; (2) past experience of receiving mental health support (formal/informal, cancer-experience specific/non-cancer experience specific) (3) preferences for support (e.g., type of support, emotional difficulties to be target, and intervention content areas); and (4) barriers and facilitators to help-seeking behavior (e.g., perceived attitudes of peers, knowledge/information, economic resources, geographical location, stigma). The interview guide will be partially informed by emerging survey results and findings from studies examining help-seeking behaviour in young adults.[32, 42, 50, 72] Interviews are estimated to last 45 to 90 minutes.

Data analysis

Statistical analysis

All statistical analyses will be conducted using SPSS V.26.0 software (IBM SPSS Statistics for Windows, V.26.0, 2019, Armonk, NY: IBM Corp.). Sociodemographic and clinical characteristics will be presented with descriptive statistics. The potential association between predicting factors as informed by Andersen´s behavioural model of health services use (predisposing, enabling, environmental, and need-related factors) and

For peer review only

the dependent variable help-seeking behaviour will be examined using univariable and multivariable logistic regressions.[72] First, univariable analyses will be conducted for each predictor separately. Variance inflation factor will be used to examine multicollinearity prior to the regression analysis. A multivariable logistic regression will be performed using stepwise manual backward selection procedure. All predictor variables will initially be included, and non-significant predictors will be eliminated with only variables significantly related to the dependent variable ($p< 0.05$) retained. For both the univariable and multivariable logistic regressions, Odds Ratios will be presented, alongside 95% confidence intervals, and p-values.

Qualitative analysis

Audio recordings will be transcribed verbatim. NVivo V.12 software (NVivo qualitative data analysis software, V.12, 2018; QSR International Pty.) will be used to assist data analysis. An inductive content analysis approach [88] will be adopted to analyse transcriptions. Only manifest content will be analysed and the following steps will be undertaken: (1) each interview transcript will be read multiple times; (2) meaning units will be identified; (3) meaning units will be labelled used descriptive codes; (4) codes will be sorted into themes and subthemes.[88] To ensure trustworthiness [89] the following strategies will be adopted: (1) two members of the research team, one researcher with experience of qualitative research and one research assistant trained in qualitative research, will analyse each interview separately with any discrepancies in analysis discussed; (2) discussion of emerging themes with the wider study team; (3) audit trails; (4) triangulation; and (5) disconfirming case analysis.[90] Further, extracts of data supporting each theme and subtheme will be presented to further improve the transparency of the analysis.[91]

Patient and public involvement

The protocol was developed without patient and public involvement (PPI). However, we will seek to involve AYACCS in pilot-testing the survey and in data interpretation of the content analysis of the embedded semi-structured interviews. Specifically, a consultation approach to PPI will be adopted [92] in pilot-testing the survey and a panel of AYACCS research partners will be asked to complete the survey (online and paper based) and comment on the feasibility, acceptability, and relevancy of the survey. Further, results of the content analysis will be presented to the panel to explore whether themes identified by the research team reflect their own experiences. The perspective of the AYACCS research partners will be incorporated into the interpretation of results and their involvement will be reported in accordance with Guidance for Reporting Involvement of Patients and the Public (GRIPP) – short form.[93]

DISCUSSION

To the best of our knowledge, this study will be the first study to examine mental health help-seeking behaviour and attitudes towards e-MH support in an AYACCS population. Results will provide an understanding of: (1) potential health service use factors and help-seeking behaviour; (2) attitudes towards e-MH; and (3) perceived need and preferences for mental health support, alongside barriers and facilitators to help seeking in the population. Our study design allows us to collect both qualitative and quantitative data and therefore will provide a more in-depth and rich understanding of mental health help-seeking behaviour and attitudes towards

For peer review only

e-MH support in an AYACCS population. The identification of factors associated with help-seeking behaviour may help identify potential targets to improve help-seeking behaviour in the future. For example, interventions could be developed to target factors found to be negatively associated with help-seeking [94] and subsequently enhance help-seeking behaviours in an AYACCS population in the future. Further, increasing our understanding of attitudes towards e-MH support and preferences regarding the provision of mental health support in the population may inform the development of more acceptable and relevant interventions for this underserved population. In addition, examining preferences for support will enable the development of future psychological interventions that are specifically tailored to and target the needs on an AYACCS population seeking support in relation to their experience of cancer.

Despite the strengths of this study, the design presents some limitations. . First, only those AYACCS who report receiving mental health support in the past six months are defined as help-seeking. However, this approach may lead to the exclusion of AYACCS who have attempted to seek formal help but not accessed. Second, the cross-sectional design does not allow us to draw any conclusions regarding cause and effect. Third, the study does not offer any compensation to participants. Research suggests compensation can facilitate the recruitment of minority populations and those from lower socioeconomic backgrounds.[95] Subsequently, this may limit the representativeness of the sample and generalizability of results, especially to marginalized young adults who may already be at an elevated risk of not seeking mental health support.[96] Forth, the current study includes a broad age range (16-39) and it may be expected that adolescents will have differing needs and preferences to young adults in their 20s or 30s. Indeed, a common limitation of research in the area is the inclusion of heterogeneous samples across different age groups.[97] However, the study is designed to be a first step towards developing more acceptable and relevant interventions for the population and may inform future research with specific AYACCS subgroups, taking into account a number of sociodemographic and clinical factors (e.g., gender, current age, age at diagnosis and cancer type). Fifth, the length of the survey may lead to respondent fatigue (e.g., lower levels of attention and motivation in later sections of the questionnaire) potentially resulting in poorer quality data or missing data. Finally, we will not evaluate response bias via comparisons of respondents versus non-responders for mailed surveys.

In conclusion, results of the study may have the potential to improve access to tailored, relevant, and acceptable mental health support for this currently underserved population.

ETHICS AND DISSEMINATION

The study will be approved by the Swedish Ethical Review Authority (Dnr: XXXX/XXX). The rights and welfare of participants will be ensured by all research being conducted in accordance with the Helsinki Declaration. Informed consent will be collected, ensuring participants are aware of requirements for study participation. Participants will be free to withdraw from the study at any time, without providing reason. Contact details for the principal investigator (coauthor LvE), the U-CARE Health and Safety Officer, and Uppsala University Data Protection Officer will be provided to all participants should there be any cause for concern regarding the conduct of the study.

For peer review only

All data will be handled according to the Patient Data Act (2008:355) and General Data Protection Regulation (EU 2016/679). Data collected via the online survey via the U-CARE-portal will be securely stored on Uppsala University servers. Data collected via the paper-based survey will be entered into an Access Database, stored on Uppsala University servers. Paper questionnaires will be stored in locked secure filing cabinets, only accessible by authorised members of the study team. Participant contact details for the interview study will be stored separately on a USB stick in a locked secure filing cabinet, separate from study data. Interviews will be audio recorded, with audio files uploaded onto and stored on Uppsala University secure server, using participant identifier numbers and immediately deleted from devices. Interview transcripts will omit any personally identifiable data and will be stored on a secure server.

Survey results will be reported in line with the Checklist for Reporting Results of Internet e-Surveys (CHERRIES) [98] and results of the embedded qualitative interview study will be reported in accordance with the Standards for Reporting Qualitative Research (SRQR).[99] Results will be published in scientific publications in peer reviewed journals and conference presentations. Lay language summaries will also be provided to all community-based organisations and support groups who support recruitment into the study.

AUTHOR'S CONTRIBUTIONS

JW: Conceptualisation, methodology, writing - original draft, supervision, project administration, funding acquisition; JM: Methodology, resources, writing - original draft, project administration; ÅC: Writing – review and editing; PE: Writing – review and editing; AH-S: Writing – review and editing; JH: Resources, writing – review and editing, project administration; MK: Methodology, resources, writing – review and editing, project administration; HR: Resources, writing – review and editing, project administration; LvE: Methodology, Writing – review and editing, supervision, funding acquisition.

FUNDING

This work is supported by U-CARE, which is a Strategic Research environment funded by the Swedish Research Council (dnr 2009–1093) and Fredrik O Ingrid Thurings Stiftelse (grant number 2018-00437).

COMPETING INTERESTS

The authors have no competing interests to declare.

COPYRIGHT AND AUTHORS' RIGHTS

I Joanne Woodford, the Submitting Author has the right to grant and does grant on behalf of all authors of the Work (as defined in the below author licence), an exclusive licence and/or a non-exclusive licence for contributions from authors who are: i) UK Crown employees; ii) where BMJ has agreed a CC BY licence shall apply, and/or iii) in accordance with the terms applicable for US Federal Government officers or employees acting as part of their official duties; on a worldwide, perpetual, irrevocable, royalty-free basis to BMJ Publishing Group Ltd ("BMJ") its licensees and where the relevant Journal is co-owned by BMJ to the co-owners of the Journal, to publish the Work in [BMJ Open] and any other BMJ products and to exploit all rights, as set out in our licence.

References

1. Gatta G, Botta L, Rossi S, et al. Childhood cancer survival in Europe 1999-2007: results of EUROCARE-5--a population-based study. *Lancet Oncol* 2014;15:35-47. doi:10.1016/S1470-2045(13)70548-5.
2. Armstrong GT, Kawashima T, Leisenring W, et al. Aging and risk of severe, disabling, life-threatening, and fatal events in the childhood cancer survivor study. *J Clin Oncol* 2014;32:1218-27. doi:10.1200/JCO.2013.51.1055.
3. Bhakta N, Liu Q, Ness KK, et al. The cumulative burden of surviving childhood cancer: an initial report from the St. Jude lifetime cohort study. *Lancet* 2017;390:2569–82. doi:10.1016/S0140-6736(17)31610-0
4. Chow EJ, Ness KK, Armstrong GT, et al. Current and coming challenges in the management of the survivorship population. *Semin Oncol* 2020;47(1):23-39. doi: 10.1053/j.seminoncol.2020.02.007.
5. Robison LL, Hudson MM. Survivors of childhood and adolescent cancer: life-long risks and responsibilities. *Nat Rev Cancer* 2014;14(1):61-70. doi:10.1038/nrc363423.
6. Winick N. Neurocognitive outcome in survivors of pediatric cancer. *Curr Opin Pediatr* 2011; 23(1):27-33. doi: 10.1097/MOP.0b013e32834255e9.
7. Kirchhoff AC, Krull KR, Ness KK, et al. Occupational outcomes of adult childhood cancer survivors: A report from the childhood cancer survivor study. *Cancer* 2011;117(13):3033-3044. doi:10.1002/cncr.25867.
8. Lancashire ER, Frobisher C, Reulen RC et al. Educational attainment among adult survivors of childhood cancer in Great Britain: a population-based cohort study. *J Natl Cancer Inst* 2010;102(4):254-270. doi:10.1093/jnci/djp498.
9. Ander M, Grönqvist H, Cernvall M, et al. Development of health-related quality of life and symptoms of anxiety and depression among persons diagnosed with cancer during adolescence: a 10-year follow-up study. *Psychooncology* 2016;25:582-89. doi:26361085.
10. Brinkman TM, Zhu L, Zeltzer LK, et al. Longitudinal patterns of psychological distress in adult survivors of childhood cancer. *Br J Cancer* 2013;109:1373-81. doi:10.1038/bjc.2013.428.
11. Seitz DC, Besier T, Debatin KM, et al. Posttraumatic stress, depression and anxiety among adult long-term survivors of cancer in adolescence. *Eur J Cancer* 2010;46:1596-06. doi:10.1016/j.ejca.2010.03.001.
12. Patton GC, Sawyer SM, Santelli JS, et al. Our future: a lancet commission on adolescent health and wellbeing. *Lancet* 2016;387:2423-78. doi:10.1016/S0140-6736(16)00579-1.
13. Blackmore SJ. Adolescence and mental health. *Lancet* 2019;393:2030-31. doi:10.1016/S0140-6736(19)31013-X.
14. Ahomäki R, Kero A, Koivisto M, et al. Purchases of antidepressants after cancer at a young age in Finland. *Int J Cancer* 2019:144:1227-33. doi:10.1002/ijc.31942 25.
15. Johannsdottir IM, Loge JH, Kiserud CE, et al. Increased prescription rates of anxiolytics and hypnotics to survivors of cancer in childhood, adolescence, and young adulthood-a population-based study. *Pediatr Blood Cancer* 2018;65:e26860. doi:10.1002/pbc.26860.
16. Brinkman TM, Ullrich NJ, Zhang N, et al. Prevalence and predictors of prescription psychoactive medication use in adult survivors of childhood cancer: a report from the childhood cancer survivor study. *J Cancer Surviv* 2013;7:104–14. doi:10.1007/s11764-012-0250-x.

For peer review only

17. Gunnes MW, Lie RT, Bjørge T, et al. Suicide and violent deaths in survivors of cancer in childhood, adolescence and young adulthood-a national cohort study. *Int J Cancer* 2017;140:575-80. doi: 10.1002/ijc.30474.
18. Bleyer A, Barr R, Ries L, etal. *Cancer in adolescents and young adults.* Springer International Publishing, 2017.
19. Bibby H, White V, Thompson K, et al. What are the unmet needs and care experiences of adolescents and young adults with cancer? A systematic review. *J Adolesc Young Adult Oncol* 2017;6:6-30. doi:10.1089/jayao.2016.0012.
20. Kaul S, Avila JC, Mutambudzi M, et al. Mental distress and health care use among survivors of adolescent and young adult cancer: a cross-sectional analysis of the national health interview survey. *Cancer* 2017;123:869-78. doi:10.1002/cncr.30417.
21. Olsson M, Jarfelt M, Pergert P, et al. Experiences of teenagers and young adults treated for cancer in Sweden. *Eur J Pncol Nurs* 2015;19:575-81. doi:10.1016/j.ejon.2015.03.003.
22. Svedberg P, Einberg EL, Wärnestål P, et al. Support from healthcare services during transition to adulthood – experiences of young adult survivors of pediatric cancer. *Eur J Oncol Nursing* 2016;21:105-12. doi:10.1016/j.ejon.2016.02.008.
23. Rebello TJ, Marques A, Gureje O, et al. Innovative strategies for closing the mental health treatment gap globally. *Curr Opin Psychiatry* 2014;27:308-14. doi:10.1097/YCO.0000000000000068.
24. Kim MA, Yi J. Life after cancer: how does public stigma increase psychological distress of childhood cancer survivors? *Int J Nurs Stud* 2014;51:1605-14. doi:10.1016/j.ijnurstu.2014.04.005.
25. Riper H, Andersson G, Christensen H, et al. Theme issue on e-mental health: a growing field in internet research. *J Med Internet Res* 2010;12:e74. doi:10.2196/jmir.1713.
26. Patel V, Saxena S, Lund C, et al. The lancet commission on global mental health and sustainable development. *Lancet* 2018;392:1553-98. doi:10.1016/S0140-6736(18)31612-X.
27. Andersson G. Internet interventions: past, present and future. *Internet Interv* 2018;12:181- 88. doi: 10.1016/j.invent.2018.03.008.
28. Bunge EL, Dickter B, Jones MK, et al. Behavioural intervention technologies and psychotherapy with youth: a review of the literature. *Curr Psychiatry Rev* 2016;12:14–28. doi:10.2174/1573400511666150930232254
29. Carlbring P, Andersson G, Cuijpers P, et al. Internet-based vs. face-to-face cognitive behaviour therapy for psychiatric and somatic disorders: an updated systematic review and meta-analysis. *Cogn Behav Ther* 2018;47:1-18. doi:10.1080/16506073.2017.1401115.
30. Sansom-Daly UM, Wakefield CE, Bryant RA, et al. Online group-based cognitive-behavioural therapy for adolescents and young adults after cancer treatment: a multicenter randomised controlled trial of recapture life-AYA. *BMC Cancer* 2012;12. doi:10.1186/1471-2407-12-339.
31. Hagström, J, Ander, M, Cernvall, et al. Heeding the psychological concerns of young cancer survivors: a single-arm feasibility trial of CBT and a cognitive behavioural conceptualization of distress. *PeerJ* 2020;8:e8714. doi:10.7717/peerj.8714.

For peer review only

32. Younes N, Chollet A, Menard E, et al. E-mental health care among young adults and help-seeking behaviours: a transversal study in a community sample. *J Med Internet Res* 2015;17:e123. doi: 10.2196/jmir.4254.

33. Ander M, Wikman A, Ljótsson B, et al. Guided internet-administered self-help to reduce symptoms of anxiety and depression among adolescents and young adults diagnosed with cancer during adolescence (U-CARE: YoungCan): a study protocol for a feasibility trial. BMJ Open 2017;7: e013906. doi:10.1136/bmjopen-2016-013906.

34. Ander M. *Cancer during adolescence - psychological consequences and development of psychological treatment.* 2017. http://uu.diva-portal.org/smash/record.jsf?pid=diva2%3A1153609&dswid=1594

35. Barnett M, McDonnell G, DeRosa A, et al. Psychosocial outcomes and interventions among cancer survivors diagnosed during adolescence and young adulthood (AYA): a systematic review. *J Cancer Surviv* 2016;10:814-31. doi:10.1007/s11764-016-0527-6.

36. Devine KA, Viola AS, Coups EJ, et al. Digital health interventions for adolescent and young adult cancer survivors. *JCO Clin Cancer Inform* 2018;2:1-15. doi:10.1200/CCI.17.00138.

37. Moody L, Turner A, Osmond J, et al. Web-based self-management for young cancer survivors: consideration of user requirements and barriers to implementation. *J Cancer Surviv* 2015;9:188–200. doi:10.1007/s11764-014-0400-4.

38. Seitz DC, Knaevelsrud C, Duran G, et al. Efficacy of an internet-based cognitive-behavioral intervention for long-term survivors of pediatric cancer: a pilot study. *Support Care Cancer* 2014;22:2075–83. doi:10.1007/s00520-014-2193-4.

39. van Ballegooijen W, Cuijpers P, van Straten A, et al. Adherence to internet-based and face-to-face cognitive behavioural therapy for depression: a meta-analysis. *PLoS One* 2014;9:e100674. doi:10.1371/journal.pone.0100674.

40. Nilsson A, Sörman K, Klingvall J, et al. MyCompass in a Swedish context - lessons learned from the transfer of a self-guided intervention targeting mental health problems. *BMC Psychiatry* 2019;19:51. doi:10.1186/s12888-019-2039-1.

41. Woodford J, Farrand P, Bessant M, et al. Recruitment into a guided internet based CBT (iCBT) intervention for depression: lesson learnt from the failure of a prevalence recruitment strategy. *Contemp Clin Trials* 2011;32:641-48. doi:10.1016/j.cct.2011.04.013.

42. Rapp AM, Chavira DA, Sugar CA, et al. Integrated primary medical-behavioural health care for adolescent and young adult depression: predictors of service use in the youth partners in care trial. *J Pediatr Psychol* 2017;42:1051-64. doi:10.1093/jpepsy/jsx057.

43. Gulliver A, Griffiths KM, Christensen H. Perceived barriers and facilitators to mental health help-seeking in young people: a systematic review. *BMC Psychiatry* 2010;10. doi:10.1186/1471-244X-10-113.

44. Kaess M, Bauer S. Promoting help-seeking using e-technology for adolescents: the ProHEAD consortium. *Trials* 2019;20:72. doi:10.1186/s13063-018-3162-x.

45. Lehmann V, Grönqvist H, Engvall G, et al. Negative and positive consequences of adolescent cancer 10 years after diagnosis: an interview-based longitudinal study in Sweden. *Psychooncology* 2014;23:1229-35. doi:10.1002/pon.3549.

For peer review only

46. Ander M, Thorsell Cederberg J, von Essen L, et al. Exploration of psychological distress experienced by survivors of adolescent cancer reporting a need for psychological support. *PLoS One* 2018;13:e0195899. doi:10.1371/journal.pone.0195899.

47. Wilford J, Buchbinder D, Fortier MA, et al. "She was a little social butterfly": a qualitative analysis of parent perception of social functioning in adolescent and young adult brain tumor survivors. *J Pediatr Oncol Nurs* 2017;34:239-49. doi:10.1177/1043454216688660.

48. Andersen RM. Revisiting the behavioral model and access to medical care: does it matter? *J Health Soc Behav* 1995;36:1-10. doi:10.2307/2137284

49. Bock JO, Hajek A, König HH. The longitudinal association between psychological factors and health care use. *Health Serv Res* 2018;53:1065-91. doi:10.1111/1475-6773.12679.

50. Gudleski GD, Satchidanand N, Dunlap LJ, et al. Predictors of medical and mental health care use in patients with irritable bowel syndrome in the United States*. Behav Res Ther* 2017;88:65-75. doi:10.1016/j.brat.2016.07.006.

51. Mills V, Van HM, Baur J, et al. Predictors of mental health service utilisation in a non-treatment seeking epidemiological sample of Australian adults. *Community Ment Health J* 2012;48:511-21. doi:10.1007/s10597-011-9439-0.

52. Mooney-Somers J, Lewis P, Kerridge I. Discursive constructions of youth cancer: findings from creative methods research with healthy young people. *J Cancer Surviv* 2016;10:427-36. doi:10.1007/s11764-015-0488-1.

53. van Schoors M, Caes L, Knoble NB, et al. Systematic review: associations between family functioning and child adjustment after pediatric cancer diagnosis: a meta-analysis. *J Pediatr Psychol* 2017;42:6-18. doi:10.1093/jpepsy/jsw070.

54. Sweeney GM, Donovan CL, March S, et al. Logging into therapy: adolescent perceptions of online therapies for mental health problems. *Internet interv* 2016;15:93-9. doi:10.1016/j.invent.2016.12.001.

55. Wetterlin FM, Mar MY, Neilson EK, et al. eMental health experiences and expectations: a survey of youths' web-based resource preferences in Canada. *J Med Internet Res* 2014;16:e29. doi:10.2196/jmir.3526.

56. Hollis C, Morris R, Martin J, et al. Technological innovations in mental healthcare: harnessing the digital revolution. *Br J Psychiatry* 2015;206:263-265. doi:10.1192/bjp.bp.113.142612.

57. Fleming T, Bavin L, Lucassen M, et al. Beyond the trial: systematic review of real-world uptake and engagement with digital self-help interventions for depression, low mood, or anxiety. *J Med Internet Res* 2018;20:e199. dio:10.2196/jmir.9275.

58. Apolinário-Hagen J, Kemper J, Stürmer C. Public acceptability of e-mental health treatment services for psychological problems: a scoping review. *JMIR Ment Health* 2017;4:e10. doi:10.2196/mental.6186.

59. van Beugen S, Ferwerda M, Hoeve D, et al. Internet-based Ccgnitive behavioral therapy for patients with chronic somatic conditions: a meta-analytic review. *J Med Internet Res* 2014;16(3):e88. doi: 10.2196/jmir.2777.

60. Radomski AD, Wozney L, McGrath P, et al. Design and delivery features that may improve the use of internet-based cognitive behavioral therapy for children and adolescents with anxiety: a realist literature

synthesis with a persuasive systems design perspective. J Med Internet Res 2019;21(2):e11128. doi: 10.2196/11128.

61. Ferwerda M, van Beugen S, van Burik A, et al. What patients think about e-health: patients' perspective on internet-based cognitive behavioral treatment for patients with rheumatoid arthritis and psoriasis. *Clin Rheumatol* 2013;32(6):869-873. doi:10.1007/s10067-013-2175-9.

62. Barakat LP, Galtieri LR, Szalda D, Schwartz LA. Assessing the psychosocial needs and program preferences of adolescents and young adults with cancer. *Support Care Cancer 2016*;24(2):823-832. doi:10.1007/s00520-015-2849-8.

63. Grönqvist H, Olsson EMG, Johansson B, et al. Fifteen challenges in establishing a multidisciplinary research program on eHealth research in university settings: a case study. *J Med Internet Res* 2017;19:e173. doi:10.2196/jmir.7310.

64. Kilsdonk E, van Dilmen-den Broeder E, van der Pal HJ, et al. Effect of web-based versus paper-based questionnaires and follow-up strategies on participation rates of Dutch childhood cancer survivors: a randomized controlled trial. *JMIR Cancer* 2015;1:e11. doi:10.2196/cancer.3905.

65. Brick JM, Williams D. Explaining rising nonresponse rates in cross-sectional surveys. *AAPSS* 2013;645:36–59. doi:10.1177/0002716212456834.

66. Batterham PJ. Recruitment of mental health survey participants using internet advertising: content, characteristics and cost effectiveness. *Int J Methods Psychiatr Res* 2014;23:184-91. doi: 10.1002/mpr.1421.

67. Gorman JR, Roberts SC, Dominick, SA. A Diversified recruitment approach incorporating social media leads to research participation among young adult-aged female cancer survivors. *J Adolesc Young Adult Oncol* 2014:3: 59–5. doi:10.1089/jayao.2013.0031.

68. O'Connor A, Jackson L, Goldsmith L, et al. Can I get a retweet please? Health research recruitment and the twittersphere. *J Adv Nurs* 2014;70:599-09. doi:10.1111/jan.12222.

69. Barnes M, Wiles N, Morrison J, et al. Exploring patients' reasons for declining contact in a cognitive behavioral therapy randomized controlled trial in primary care. *Br J Gen Pract* 2012;62:e371–77. doi: 10.3399/bjgp12X641492.

70. Woodford J, Wikman A, Cernvall M, et al. Study protocol for a feasibility study of an internet-administered, guided, CBT-based, self-help intervention (ENGAGE) for parents of children previously treated for cancer. *BMJ Open* 2018;8:e023708. doi:10.1136/bmjopen-2018-023708.

71. Kilsdonk E, Wendel E, van Dulmen-den Broeder E, et al. Participation rates of childhood cancer survivors to self-administered questionnaires: a systematic review. *Eur J Cancer Care* 2017;26:e12462. doi:10.1111/ecc.12462.

72. Boerema AM, Kleiboer A, Beekman ATet al. Determinants of help-seeking behavior in depression: a cross-sectional study. *BMC Psychiatry* 2016;16. doi:10.1186/s12888-016-0790-0.

73. Apolinário-Hagen J, Vehreschild V, Alkoudmani RM. Current views and perspectives on e-mental health: an exploratory survey study for understanding public attitudes toward internet-based psychotherapy in Germany. *JMIR Ment Health* 2017;4:e8. doi:10.2196/mental.6375.

74. Apolinário-Hagen J, Harrer M, Kählke F, et al. Public attitudes toward guided internet-based therapies: web-based survey study. *JMIR Ment Health* 2018;5:e10735. doi:10.2196/10735.

For peer review only

For peer review only - http://bmjopen.bmj.com/site/about/guidelines.xhtml

75. Alfonsson S, Wallin E, Maathz P. Factor structure and validity of the depression, anxiety and stress scale-21 in Swedish translation. *J Psychiatr Ment Health Nurs* 2017;24:154-62. doi:10.1111/jpm.

76. Brown TA, Chorpita BF, Korotitsch W, et al. Psychometric properties of the depression anxiety stress scales (DASS) in clinical samples. *Behav Res Ther* 1997;35:79-89. doi:10.1016/s0005-7967(96)00068-x.

77. Ng F, Trauer T, Dodd S, et al. The validity of the 21-item version of the depression anxiety stress scales as a routine clinical outcome measure. *Acta Neuropsychiatr* 2007;19:304-10. doi:10.1111/j.1601-5215.2007.00217.x.

78. Sinclair SJ, Siefert CJ, Slavin-Mulford JM, et al. Psychometric evaluation and normative data for the depression, anxiety, and stress scales-21 (DASS-21) in a nonclinical sample of U.S. adults. *Eval Health Prof* 2012;35:259-79. doi:10.1177/0163278711424282.

79. Bottesi G, Ghisi M, Altoè G, etal. The Italian version of the depression anxiety stress scales-21: factor structure and psychometric properties on community and clinical samples. *Compr Psychiatry* 2015;60:170-81. doi: 10.1016/j.comppsych.2015.04.005.

80. Komiya N, Good GE, Sherrod NB. Emotional openness as a predictor of college students' attitudes toward seeking psychological help. *J Couns Psychol* 2000;47:138–43. doi:10.1037/AJ022-0167,47.1.138

81. Bathje GJ, Pryor JB. The relationships of public and self-stigma to seeking mental health services. *J Ment Health Couns* 2011;33:161–76. doi:10.17744/mehc.33.2.g632039274160411.

82. Pinto MD, Greenblatt AM, Hickman RL, et al. Assessing the critical parameters of eSMART-MH: a promising avatar-based digital therapeutic intervention to reduce depressive symptoms. *Perspect Psychiatr Care* 2016;52:157-68. doi:10.1111/ppc.12112.

83. Cohen S, Mermelstein R, Kamarck T, et al. Measuring the functional components of social support. In: Sarason IG, Sarason BR, eds. *Social support: theory, research, and applications*. Martinus Nijhoff 1985. 73–94.

84. Jung H, von Stenberg K, Davis K. Expanding a measure of mental health literacy: development and validation of multicomponent mental health literacy measure. *Psychiatry Res* 2016;243:278-86. doi:10.1016/j.addr.2016.07.002.

85. Fusch PI, Ness LR. Are we there yet? Data saturation in qualitative research. *Qual Rep* 2015;20:1408-1416.

86. Guest G, Bunce A, Johnson L. How many interviews are enough? An experiment with data saturation and variability. *Sage journal* 2006;18:59-82. doi:10.1177/1525822X05279903.

87. Sim J., Saunders B, Waterfield J, Kingstone T. Can sample size in qualitative research be determined a priori? *Int J Soc Res Methodol* 2018; 21:5, 619-634. doi: 10.1080/13645579.2018.1454643.

88. Elo S, Kyngäs H. The qualitative content analysis process. *J Adv Nurs*. 2008;62:107-15. doi:10.1111/j.1365-2648.2007.04569.x.

89. Krefting L. Rigor in qualitative research: the assessment of trustworthiness. *Am J Occup Ther* 1991;45:214-22. doi:10.5014/ajot.45.3.214 [published Online First: 1 March 1991].

90. Patton MQ. Enhancing the quality and credibility of qualitative analysis. *Health Serv Res* 1999;34:1189–1208.

91. Elliott R, Fischer CT, Rennie DL. Evolving guidelines for publication of qualitative research studies in psychology and related fields. *Br J Clin Psychol*. 1999;38:215-29. doi:10.1348/014466599162782.

For peer review only

92. Jennings H., Slade M., Bates P. et al. Best practice framework for patient and public involvement (PPI) in collaborative data analysis of qualitative mental health research: methodology development and refinement. *BMC Psychiatry*. 2018:18. doi:10.1186/s12888-018-1794-8.

93. Staniszewska S, Brett J, Simera I, et al. GRIPP2 reporting checklists: tools to improve reporting of patient and public involvement in research. *BMJ* 2017;358:j3453. doi:10.1136/bmj.j3453.

94. Kauer SD, Mangan C, Sanci L. Do online mental health services improve help-seeking for young people? A systematic review. *J Med Internet Res* 2014;16:e66. doi:10.2196/jmir.3103.

95. Kost RG, de Rosa JC. Impact of survey length and compensation on validity, reliability, and sample characteristics for Ultrashort-, Short-, and Long-Research Participant Perception Surveys. *J Clin Transl Sci*. 2018;2(1):31-37. doi:10.1017/cts.2018.18.

96. Sapiro, B., Ward, A. Marginalized youth, mental health, and connection with others: a review of the literature. *Child Adolesc Soc Work* 2020; J37:343–357. doi.10.1007/s10560-019-00628-5.

97. Warner EL, Kent EE, Trevino KM, et al. Social well-being among adolescents and young adults with cancer: a systematic review. *Cancer* 2016;122(7):1029-1037. doi:10.1002/cncr.29866.

98. Eysenbach G. Improving the quality of web surveys: the checklist for reporting results of internet e-surveys (CHERRIES). J Med Internet Res 2004;6:e34. doi:10.2196%2Fjmir.6.3.e34.

99. O'Brien BC, Harris IB, Beckman TJ, et al. Standards for reporting qualitative research: a synthesis of recommendations. *Academic Medicine* 2014;89:1245-51. doi:10.1097/ACM.0000000000000388.

For peer review only

BMJ Open

Help-seeking behaviour and attitudes towards internet-administered psychological support among adolescent and young adults previously treated for cancer during childhood: Protocol for a survey and embedded qualitative interview study in Sweden

Journal:	*BMJ Open*
Manuscript ID	bmjopen-2020-041350.R2
Article Type:	Protocol
Date Submitted by the Author:	05-Mar-2021
Complete List of Authors:	Woodford, Joanne; Uppsala University, Clinical Psychology in Healthcare, Department of Women´s and Children´s Health<br>Månberg, Jenny; Region Vasternorrland, Child and Adolescent Psychiatry<br>Cajander, Åsa; Uppsala University, Department of Information Technology<br>Enebrink, Pia; Karolinska Institute, Department of Clinical Neuroscience<br>Harila-Saari , Arja; Uppsala University, Pediatric Oncology, Department of Women's and Children's Health,<br>Hagström, Josefin; Uppsala University, Clinical Psychology in Healthcare, Department of Women's and Children's Health<br>Karlsson, Mathilda; Uppsala University, Clinical Psychology in Healthcare, Department of Women´s and Children´s Health<br>Placid Solimena, Hanna; Uppsala University, International Maternal and Child Health Care, Department of Women's and Children's Health<br>von Essen, Louise; Uppsala University, Clinical Psychology in Healthcare, Department of Women's and Children's Health
**Primary Subject Heading**:	Mental health
Secondary Subject Heading:	Oncology
Keywords:	MENTAL HEALTH, Paediatric oncology < ONCOLOGY, PRIMARY CARE, Child & adolescent psychiatry < PSYCHIATRY, QUALITATIVE RESEARCH

SCHOLARONE™ Manuscripts

I, the Submitting Author has the right to grant and does grant on behalf of all authors of the Work (as defined in the below author licence), an exclusive licence and/or a non-exclusive licence for contributions from authors who are: i) UK Crown employees; ii) where BMJ has agreed a CC-BY licence shall apply, and/or iii) in accordance with the terms applicable for US Federal Government officers or employees acting as part of their official duties; on a worldwide, perpetual, irrevocable, royalty-free basis to BMJ Publishing Group Ltd ("BMJ") its licensees and where the relevant Journal is co-owned by BMJ to the co-owners of the Journal, to publish the Work in this journal and any other BMJ products and to exploit all rights, as set out in our licence.

The Submitting Author accepts and understands that any supply made under these terms is made by BMJ to the Submitting Author unless you are acting as an employee on behalf of your employer or a postgraduate student of an affiliated institution which is paying any applicable article publishing charge ("APC") for Open Access articles. Where the Submitting Author wishes to make the Work available on an Open Access basis (and intends to pay the relevant APC), the terms of reuse of such Open Access shall be governed by a Creative Commons licence – details of these licences and which Creative Commons licence will apply to this Work are set out in our licence referred to above.

Other than as permitted in any relevant BMJ Author's Self Archiving Policies, I confirm this Work has not been accepted for publication elsewhere, is not being considered for publication elsewhere and does not duplicate material already published. I confirm all authors consent to publication of this Work and authorise the granting of this licence.

For peer review only

Help-seeking behaviour and attitudes towards internet-administered psychological support among adolescent and young adults previously treated for cancer during childhood: Protocol for a survey and embedded qualitative interview study in Sweden

Joanne WOODFORD*[1] (joanne.woodford@kbh.uu.se), Jenny MÅNBERG[1,2] (jenny.manberg@rvn.se), Åsa CAJANDER[3] (asa.cajander@it.uu.se), Pia ENEBRINK[4] (pia.enebrink@ki.se), Arja HARILA-SAARI[5] (arja.harila-saari@kbh.uu.se), Josefin HAGSTRÖM[1] (josefin.hagstrom@kbh.uu.se), Mathilda KARLSSON[1] (mathilda.karlsson@kbh.uu.se), Hanna PLACID SOLIMENA[6] (hannaplacid@gmail.com), & Louise VON ESSEN[1] (louise-von.essen@kbh.uu.se).

Corresponding author

Dr Joanne Woodford, Clinical Psychology in Healthcare, Department of Women's and Children's Health. Uppsala University Dag Hammarskjölds väg 14B, 751 05 Uppsala, Sweden. E-mail: joanne.woodford@kbh.uu.se Telephone: 018-471-65 73

Affiliation

[1]Clinical Psychology in Healthcare, Department of Women´s and Children´s Health, Uppsala University, Uppsala, Sweden

[2]Child and Adolescent Psychiatry, Region Västernorrland, Sweden

[3]Visual Information and Interaction, Department of Information Technology, Uppsala University, Uppsala, Sweden

[4]Department of Clinical Neuroscience, Division of Psychology, Karolinska Institute, Stockholm, Sweden

[5]Pediatric Neurology / Pediatric Oncology, Department of Women's and Children's Health, Uppsala University, Uppsala, Sweden

[6]International Maternal and Child Health Care, Department of Women's and Children's Health, Uppsala University, Uppsala, Sweden

Word count: 5115

For peer review only

ABSTRACT

Introduction: A subgroup of adolescent and young adult childhood cancer survivors (AYACCS) are at increased risk of psychological distress. Despite this, AYACCS experience difficulties accessing psychological support. E-mental health (e-MH) may offer a solution to reduce this treatment gap. However research examining e-MH for AYACCS has experienced difficulties with recruitment, retention, and adherence. Such difficulties may relate to: (1) help-seeking behaviour and/or (2) e-MH acceptability. The overall study aims are to: (1) examine potential associations between health service use factors, informed by Andersen´s behavioural model of health services use, and help-seeking behaviour; (2) examine attitudes towards e-MH interventions; and (3) explore perceived need for mental health support; past experience of receiving mental health support; preferences for support; and barriers and facilitators to help-seeking.

Methods and analysis: An online and paper-based cross-sectional self-report survey (98 items) and embedded qualitative interview study across Sweden, with a target sample size of n=365. Participants are 16-39 years, diagnosed with cancer when 0-18 years, and have completed successful cancer treatment. The survey examines: sociodemographic and clinical characteristics, actual help-seeking behaviour, attitudes towards e-MH, stigma of mental illness, mental health literacy, social support, and current symptoms of depression, anxiety, and stress. Survey respondents with past and/or current experience of mental health difficulties are invited into the qualitative interview study to explore: (1) perceived need for mental health support; (2) past experience of receiving mental health support; (3) preferences for support; and (4) barriers and facilitators to help-seeking. Potential associations between health service use factors and help-seeking behaviour are examined using bivariate analyses and multivariable multinomial logistic regressions. Qualitative interviews are analysed using content analysis.

Ethics and dissemination: Ethical approval has been obtained from the Swedish Ethical Review Authority (Dnr: 2020-06271). Results will be disseminated in scientific publications and academic conference presentations.

Trial registration number: ISRCTN70570236

For peer review only

STRENGTHS AND LIMITATIONS OF THIS STUDY

- The mixed methods design may aid a rich and insightful understanding of the investigated phenomena.
- Results may be used to inform the development of acceptable, relevant, and accessible mental health interventions for adolescent and young adult childhood cancer survivors.
- Reasons for not receiving a mental health intervention are not explored and only those who report receiving support in the past six months are defined as help-seeking. This approach may exclude adolescent and young adult childhood cancer survivors who have attempted to seek formal help (help-seeking) but did not receive help.
- No conclusions concerning cause and effect can be drawn given the cross-sectional design.

For peer review only

INTRODUCTION

Medical improvements have resulted in an overall five-year survival rate of 81.2 % from cancer diagnosed during childhood (0-18 years) in Northern Europe.[1] However, significant challenges, such as late effects, remain after treatment completion.[2,3] Survivors of childhood cancer report a number of long-term adverse physical and developmental outcomes [4-6] and social and economic impacts, including poorer educational and occupational achievements.[7,8] A further common late effect is symptoms of psychological distress, for example, posttraumatic stress, anxiety, and depression, symptoms of which have been found to persist decades post diagnosis.[9-11] Difficulties with psychological distress are further complicated given that adolescence is a period of increased vulnerability to mental health problems [12] with difficulties such as depression and anxiety commonly first appearing before 24 years of age.[13] Indeed, individuals diagnosed with cancer during childhood, adolescence, and young adulthood have been found to be at an increased likelihood of being prescribed hypnotics and anxiolytics [14,15] of using antidepressant medication over the long-term,[16] and are at an elevated risk of suicide.[17] As such, adolescent and young adult childhood cancer survivors (AYACCS), commonly defined as cancer survivors diagnosed during childhood currently aged between 15-39 years,[18] are particularly vulnerable to experiencing adverse psychological and social outcomes, and there is a significant need for long-term care and support.[5] However, despite a clear need for the provision of mental health support, a mental health treatment gap remains, with few AYACCS being offered professional psychological support.[19-22] Reasons for this treatment gap may pertain to a lack of qualified healthcare professionals, poor service provision, geographical barriers,[23] and public stigma (e.g., the general public holding negative beliefs about cancer survivors, for example, being less competent).[24]

E-mental health (e-MH), defined as the provision of psychological support via information technology and new media (e.g., online, smartphone applications, videoconferencing),[25] may represent a solution to reduce the mental health treatment gap.[26] One example of e-MH is internet-administered cognitive behavioural therapy (ICBT). ICBT has been found to be effective for a range of common mental health difficulties, such as depression and anxiety,[27] including for young adult populations [28] and has been found to be as effective as traditional face-to-face CBT.[29] E-MH interventions may help overcome practical barriers associated with accessing traditional face-to-face psychological support (e.g., geographical, financial, and time-related barriers) [30] that have been observed within the AYACCS population.[31] Further, e-MH is associated with anonymity

and privacy [32] thus potentially overcoming barriers associated with public stigma. Indeed, research has identified that the provision of e-MH interventions may be a potential solution for the AYACCS population.[33-38] However, research examining e-MH support for an AYACCS population has encountered challenges with recruitment, attrition, and internet use.[34,38] For example, one study examining the feasibility and acceptability of e-MH recruited only 28 participants over a 12 month period and experienced almost 30% attrition.[38] Another feasibility study of e-MH for adolescents and young adults who had experienced cancer during the adolescent period recruited only 6 participants from a total of 320 potential participants invited into the study, and experienced a 100% attrition rate.[34] Indeed, a recent review of e-Health interventions for AYACCS concluded that engaging the population was challenging, and there is currently a lack of literature concerning how to better engage the population.[36] However, challenges with recruitment, attrition, and internet use are common in the field of e-MH research.[39-41] Potentially, challenges with recruitment, attrition, and internet use may be related to help-seeking behaviour and the acceptability of e-MH for the population.

Help-seeking behaviour

Low rates of formal help-seeking behaviour for mental health difficulties is common amongst an adolescent and young adult population in general.[42] A number of potential barriers to help-seeking have been identified, including stigma, embarrassment, and poor mental health literacy.[43, 44] Indeed, AYACCS report difficulties with stigma and alienation in relation to the cancer disease [45] and as such may be reluctant to identify themselves as experiencing mental health difficulties. Further, whilst social support has been found to facilitate help-seeking behaviour for mental health difficulties in adolescent and young adult populations,[43] AYACCS commonly report difficulties with social withdrawal and loneliness.[46, 47] However, at present there is a lack of research concerning help-seeking behaviour for mental health difficulties experienced by an AYACCS population.

A frequently used approach for identifying factors associated with help-seeking is Andersen´s behavioural model of health services use behavior.[48] The model hypothesizes a number of factors to be associated with help-seeking behaviour: (1) predisposing factors including sociodemographic variables (e.g., age and gender) and health related beliefs and knowledge;[49] (2) enabling factors relating to logistical elements to receiving care and resources (e.g., financial factors and social support);[50] (3) external environmental factors (e.g., location); and (4) need-related factors (e.g., symptoms of psychological distress). Recently the model has been adopted to examine factors associated with accessing both traditional and e-MH services within a young adult (18-39) population.[32] Interestingly, the study identified a weaker utilisation of e-MH by young people who had experienced negative childhood experiences during childhood (e.g., family conflict, bullying, or lack of affection) [32]. This finding is of particular importance seeing that negative experiences of childhood are normally associated with increased mental healthcare utilisation.[51] Further, given evidence to suggest some AYACCS may experience bullying related to their cancer experience [52] and poor family functioning for example conflict [53] it may be important to examine whether negative childhood experiences are associated with reduced mental healthcare utilisation in an AYACCS population. Further, to the best of our knowledge, Andersen´s behavioural model of health services use has not yet been applied to an AYACCS population and factors associated with help-seeking in the population remain unclear.

For peer review only - http://bmjopen.bmj.com/site/about/guidelines.xhtml

Acceptability

Whilst adolescents appear to consider e-MH interventions to be potentially acceptable they still report a preference for face-to-face over e-MH interventions.[54] Indeed, there is some indication e-MH resources are not widely accessed by young people.[55] One potential difficulty may be related to what has been coined the '*technology push'*, whereby e-MH solutions are designed to fit available technology, rather than to meet the unmet needs and preferences of end-users.[56] Further, research indicates poor uptake and ongoing use of e-MH interventions that have been implemented into healthcare settings within the general population.[57] As such, there is a need to understand preferences and attitudes towards e-MH interventions within specific populations, via both quantitative and qualitative research, to inform future e-MH developments.[58] To the best of our knowledge, there has been no exploration to date of the attitudes and preferences towards e-MH interventions within an AYACCS population. In addition, tailoring e-MH interventions to specific patient groups has been associated with increased effectiveness [59] and adherence.[60] Indeed, rates of attrition may be reduced, and adherence increased, if the perspective of the population using the intervention is taken into account when developing e-MH interventions.[61] Further, research into the psychological needs of an AYACCS population indicates a need for increased focus on the specific psychological and psychosocial needs of the population to enable to provision of more tailored support to the cancer experience.[62] As such, there is need to explore AYCACCs general preferences for psychological support, including potential intervention content.

Study aims and research questions

The overall aims of the study are to: (1) examine potential associations between health service use factors, informed by Andersen´s behavioural model of health services use, and help-seeking behaviour; (2) examine attitudes towards e-MH interventions; and (3) explore perceived need and past experiences of mental health support; preferences for support; and barriers and facilitators to help-seeking. Specifically, the following research questions will be examined:

(1) Are there associations between predisposing, enabling, environmental, and needs related health service use factors, informed by Andersen´s behavioural model of health, and help-seeking behaviour within an AYACCS population?

(2) What attitudes are held by an AYACCS population towards e-MH interventions?

(3) What is the perceived need for mental health support within an AYACCS population?

(4) What are AYACCS experiences of past receipt of cancer specific and non-cancer experience specific mental health support?

(5) What preferences do an AYACCS population hold towards mental health support related to their cancer experience?

(6) What are the barriers and facilitators do an AYACCA population experience seeking help for mental health support related to their cancer experience?

METHOD AND ANALYSIS

Study design

For peer review only

An online and paper-based, cross-sectional, self-report survey and embedded qualitative interview study across Sweden. The study has been registered in the ISRCTN clinical trial registry (ISRCTN70570236).

Eligibility criteria

Eligible participants will: (1) be an adolescent or young adult, aged 16-39 years, at study start; (2) have been diagnosed with childhood cancer when 0-18 years; (3) have completed successful cancer treatment (including relapses) at a minimum 3 months ago according to self-report; (4) be able to read and write in Swedish; and (5) currently reside in Sweden.

Recruitment

A multifaceted approach to recruitment will be adopted, using two main strategies:

Swedish Childhood Cancer Registry

Personal identification numbers of AYACCS will be obtained from the Swedish Childhood Cancer Registry (National Quality Registry, initiated in 1982). Next, addresses will be obtained via NAVET, a population registry held by the Swedish Tax Agency. NAVET is continuously updated with important information about the Swedish population, including deaths and can, as such, provide current addresses and information concerning the vital status of AYACCS. The Swedish Tax Agency will provide this information via a secure online file transfer system (e-transport) on the same day information is requested by the study team. Study invitation letters will be sent to AYACCS on the same day that information is provided via e-transport in order to minimise the likelihood of inviting a deceased AYACCS. AYACCS will be invited to participate by the study team on a weekly basis, using blocks of 100 until the target sample size has been reached. Blocks will be selected randomly, with minimisation used to ensure balance between factors that may affect outcomes: sex (male; female); current age (16-24; 25-39); and age at diagnosis (0-12; 13-18).

Study invitation packs sent to home addresses will include a study invitation letter, study information, a link to the online survey via the U-CARE-portal (www.u-care.se), a secure internet research platform to support data collection and provision of e-MH interventions [63] and a study log-in code. All potential participants identified via the Swedish Childhood Cancer Registry and invited via mail-out will be provided with a link to the online survey on the U-CARE-portal and a study log-in code. In addition, given evidence to suggest the provision of choice between web-based and paper-based surveys can increase survey response rates amongst childhood cancer survivors,[64] we will include a paper-based survey and freepost envelope with each invitation letter.

Technology-based approaches

Considering that response rates to mail-out surveys have declined over the past two decades [65] we will also utilise technology-based recruitment approaches shown to be effective.[66] Indeed, social media recruitment is successful in recruiting young adult cancer survivor (aged 20–35 years) populations.[67] Specifically, we will identify community-based organisations and support groups with an online presence for AYACCS in Sweden. Examples may include: the Swedish Cancer Foundation and Childhood Cancer Foundation; and Young Cancer. Organisations and support groups will be asked to place advertisements on their websites; distribute

For peer review only

advertisements via e-mail lists and e-newsletters; and post advertisements on social networking sites (e.g., Facebook, Twitter, and Instagram).

Each online advertisement will include brief study information, a link to the online survey on the U-CARE-portal, and a study log-in code unique to each organisation and technologically-based recruitment type to allow for the examination of success of each technology-based recruitment strategy. Organisations and support groups utilising social media will be encouraged to re-post the advertisement at regular intervals following evidence suggesting that re-posting is required to meet recruitment targets.[68]

Sample size estimation

The Swedish Childhood Cancer Registry has some 9,880 Swedish persons diagnosed when 0-18 years registered since 1982. However, some will have been classified as having a benign tumor, some have been registered twice, and some will be deceased. As such, the number of potentially eligible cases will be lower and estimated to be approximately 5,745. Sample size calculation indicated that a minimum of 365 participants would be required, with a power of 0.9 and $p<.05$ (two-tailed). Assuming a response rate of approximately 40%, as indicated by a systematic review of participation rates in self-administered questionnaire studies with childhood cancer survivors [69] we estimate inviting 913 potential participants to achieve the required sample size.

Procedure

Online survey

Potential participants accessing the survey via the U-CARE-portal will be presented with study information in both text and video format. Participants will not be offered any financial reimbursements for taking part in the survey. Those interested will be able to log into the U-CARE-portal using a study log-in code and will need to identify themselves via mobile telephone and/or Bank ID (a citizen authentication system). Potential participants will then be able to provide informed consent online and will be presented with brief screening questions asking for: (1) date of birth; (2) age when diagnosed with cancer; (3) date of last cancer treatment completed; (4) able to read and write in Swedish (yes/no); and (5) whether currently residing in Sweden (yes/no). Eligible participants will be provided access to the online survey (see Outcomes). The online survey will be open for a period of six weeks. Each item completed in the survey is auto-saved and participants are also able to select "save and continue later" if they wish to return to the survey before submitting. Upon completion of the survey a purposive sample of participants with past and/or present experience of mental health difficulties will be invited to take part in an interview (see Embedded qualitative interview study for further details). These participants will be provided with study information and online consent form, as well as asked to provide contact details.

Paper-based survey

Potential participants receiving study invitation via mail-out will be able to complete a paper-based survey. The survey includes a written consent form and brief screening questions as the online survey. Participants can return the paper-based survey to the study team using the freepost envelope provided in the study invitation

For peer review only

pack. In addition, participants will be asked to provide consent if they agree to be contacted about participation in an interview study.

Reminders

Reminder study invitation packs will be resent at 2 weeks and 6 weeks after sending the initial study invitation to AYACCS who do respond to the study invitation. It will be clearly stated in the study information sheet, that reminder letters will be sent to non-responders. In addition, participants who consent to complete the online survey will receive reminders via SMS and email to complete the survey at 4 weeks, 2 weeks, and 2 days prior to the survey closing.

Survey items

An online and paper-based survey, consisting of 98 items in Swedish. The survey comprises eight subsections: (1) sociodemographic characteristics (11 items); (2) clinical cancer related characteristics (3 items); (3) actual help-seeking behaviour (2 items); (4) attitudes towards internet-administered interventions (17 items); (5) stigma of mental illness (5 items); (6) mental health literacy (26 items); (7) social support (12 items) and; (8) current symptoms of depression, anxiety, and stress (21 items). One open question at the end of the survey offers and opportunity provide any further information concerning emotional distress and preferences for support after childhood cancer. Potential predisposing, enabling, environmental, and need-related factors examined with the survey are summarised in Table 1.

Sociodemographic characteristics

Data on the following background and sociodemographic characteristics will be collected: (1) sex; (2) relationship status; (3) having children or not; (4) Swedish county of residence; (5) living in urban or rural area; (6) level of education; (7) employment status; (8) place of birth; (9) mother's place of birth; (10) father's place of birth; and (11) childhood negative events (bullying, family conflict, lack of affection).

Clinical cancer related characteristics

The following clinical characteristics related to cancer will be collected: (1) type of cancer (leukemia, central nervous system tumor, solid tumor, lymphoma or other malignancy); (2) number of relapses (zero, one or more than one); and (3) current late effects of cancer (multiple choice listed).

Actual help-seeking behaviour

The following items will be used to examine actual help-seeking behavior: (1) emotional health difficulties related to the childhood cancer experience; (2) the type of support received by those who accessed support in the past 6 months, and/or in the past (not including the past 6 months): (a) face-to-face individual or group support from mental health professional; (b) face-to-face support from a general practitioner/family doctor; (c) technology-administered mental health support (e.g., online therapy programme, smartphone application, video or teleconferencing); (d) technology-administered support from a general practitioner/family doctor (e.g., online, video, or teleconferencing); (e) social media support group; (f) phone help-line; (g) family, partner, or friend/s; (h) religious leader; and (i) other. Type of support will be categorized into: (1) face-to-face professional support;

For peer review only

(2) technology-administered professional support; and (3) informal support (social media support group, family, partner, friends, and religious leader). Those who sought support for mental health difficulties in the past 6 months and received support from a health professional either face-to-face or technology assisted will be defined as 'help-seeking'.[70]

Attitudes towards internet-administered interventions

The 17-item e-Therapy Attitudes Measure [ETAM: 71,72] will be adopted to measure attitudes towards internet-administered interventions, however, for the purpose of the present certain one item concerning health insurance companies was excluded due to not being relevant in the Swedish context. The questionnaire begins with definitions of three types of internet-administered interventions: (1) unguided internet-administered self-help programs; (2) therapist guided internet-administered self-help programs; and (3) psychological therapy delivered via videoconferencing. Subsequently, respondents are required to state which type of internet-administered intervention they would prefer, or state if they would not use internet-administered treatment at all. Subsequently, the ETAM is presented and explores attitudes towards internet-administered interventions. Specifically, respondents are instructed to state whether they agree with each statement on a 5-point rating scale ranging from 0 ("strongly disagree") to 4 ("strongly agree") and are asked to rate items considering therapist guided internet-administered self-help programs. The ETAM has been demonstrated to have good internal-consistency reliability ($\alpha = 0.89$).[72]

Symptoms of depression, anxiety, and stress

The Swedish version [73] of the (7-item depression, 7-item anxiety, and 7-item stress subscales) Depression Anxiety and Stress Scale [DASS-21: 74,75] will be adopted to examine symptoms of depression, anxiety, and stress. The DASS-21 has adequate psychometric properties [76] and satisfactory test-retest reliability,[77] with support for the factor structure and convergent validity of the Swedish version.[73]

Stigma of mental illness

The 5-item Stigma Scale for Receiving Psychological Help [SSRPH: 78] will be adopted to measure public stigma, i.e. negative perceptions concerning the receipt of mental health services. The measure has demonstrated to be reliable and valid in studies with young adults [79] and adolescents.[80] Respondents rate degrees of agreement using a four-point Likert scale ranging from 0 ("strongly disagree") to 3 ("strongly agree"), with a higher score representing a higher level of stigma.

Social support

The 12-item Interpersonal Support Evaluation List [ISEL-12: 81] will be adopted to examine perceptions of social support. Responses are provided on a four-point Likert scale, ranging from 1 ("definitely false") to 4 ("definitely true").

Mental health literacy (MHL)

A 26-item multicomponent MHL measure, demonstrated to have good internal consistency (of 0.83 of the KR-20 coefficient = 0.83),[82] and will be adopted to assess knowledge-oriented, beliefs-oriented, and resource-

For peer review only

oriented MHL. The first 22 items examining knowledge-orientated and beliefs-orientated MHL are answered on a five-point Likert scale ranging from 0 (“strongly disagree”) to 4 (“strongly agree”) and the option of “I don’t know.” The response format for the final 4 items, examining resource-orientated MHL, is “yes” or “no”.

Table 1: Factors to be examined as per Andersen´s behavioural model of health services use

**Andersen´s behavioural model of health services use**	**Factors***
Predisposing	Sex (male/female/other)<br>Age (16-24/25-39)<br>Time since first cancer treatment (years)<br>Place of birth (born in Sweden/born outside of Sweden)<br>Childhood negative events (yes/no)<br>Level of education (≤upper secondary school/> upper secondary school)<br>Public stigma (SSRPH)<br>Mental health literacy (MHL)
Enabling	Type of cancer (leukemia/CNS tumor/solid tumor/lymphoma/other)<br>Relationship status<br>Employment status (yes/no/studying)<br>Social support (ISEL-12)
Need-related	Symptoms of depression (DASS-21 depression subscale)<br>Symptoms of anxiety (DASS-21 anxiety subscale)<br>Symptoms of stress (DASS-21 stress subscale)<br>Self-reported experience of mental health difficulties in past 6 months (yes/no)
Environmental	Region of Sweden (South/Mid/North)<br>Rural or urban area
Actual help-seeking behaviour (dependent variable)	Receipt of mental health support in the past 6 months Type of mental health support received in the past 6 months (formal/technology/informal)

Footnote: *Outcomes selected informed by previous research [32, 42, 50, 70]

Translation of English language outcome measurements into Swedish

Currently, there are no Swedish versions of the following outcome measurements: (1) ETAM, (3) SSRPH, (4) ISEL, and (5) MLH. The following steps will be taken to translate: (1) one clinical psychologist, native Swedish speaker with advanced knowledge of English, will translate each outcome measurement to Swedish; and (2) translations will be reviewed, discussed, and revised with a research assistant in our study team who is a native Swedish speaker with advanced knowledge of English. Professional back translation to English and discussion

For peer review only

with the original author is out of scope for the present study due to time and resource limitations. However, permission to translate outcome measurements to Swedish will be sought.

Embedded qualitative interview study

An embedded qualitative interview study will be conducted to explore: (1) perceived need for mental health support; (2) past experience of receiving mental health support; (3) preferences for support; and (4) barriers and facilitators to help-seeking. As this study will be examining mental health help-seeking, only respondents meeting the following criteria will be invited into the study: (1) self-reported current and/or past experience of mental health difficulties during life time (yes); and/or (2) DASS-21 depression score ≥5 and/or (3) DASS-21 anxiety score ≥4 and/or DASS-21 stress score ≥8.Respondents will be provided with full study information and an online consent form. Respondents meeting these criteria will be invited consecutively until thematic data saturation is met.[83, 84] As the decision concerning whether data saturation has been met is made during the analytic process it is difficult to determine the number of interviews a priori, however we may anticipate interviewing approximately 20-30 participants.[85] As we are interested in both barriers and facilitators to seeking help, we will endeavour to interview participants classified as help-seekers and non-help-seekers. For participants who send the consent form and reply slip via the post, those meeting the inclusion criteria will be contacted by the study team to provide full study information and obtain informed consent. Interviews will be conducted either via the telephone or a secure video-conferencing system on the U-CARE-portal. Interviews will be audio recorded with informed consent and transcribed with personal information removed. Participants will be informed about the recording and give approval when giving consent to the interview. An interview guide will be developed, consisting of open-ended questions, structured around the third research objective covering the following topics: (1) perceived need for mental health support; (2) past experience of receiving mental health support (formal/informal, cancer-experience specific/non-cancer experience specific) (3) preferences for support (e.g., type of support, emotional difficulties to be target, and intervention content areas); and (4) barriers and facilitators to help-seeking behavior (e.g., perceived attitudes of peers, knowledge/information, economic resources, geographical location, stigma). The interview guide will be partially informed by emerging survey results and findings from studies examining help-seeking behaviour in young adults.[32, 42, 50, 70] Interviews are estimated to last 45 to 90 minutes.

Data analysis

Statistical analysis

All statistical analyses will be conducted using SPSS V.26.0 software (IBM SPSS Statistics for Windows, V.26.0, 2019, Armonk, NY: IBM Corp.). Sociodemographic and clinical characteristics will be presented with descriptive statistics. The potential association between predicting factors as informed by Andersen´s behavioural model of health services use (predisposing, enabling, environmental, and need-related factors) and the dependent variable help-seeking behaviour e.g., receipt of mental health support in the past 6 months will be examined using univariable and multivariable logistic regressions.[70] The non-help-seeking group will used as the reference group.[70] First, univariable analyses will be conducted for each predictor separately. Variance inflation factor will be used to examine multicollinearity prior to the regression analysis. A multivariable logistic regression will be performed using stepwise manual backward selection procedure. All predictor

For peer review only

variables will initially be included, and non-significant predictors will be eliminated with only variables significantly related to the dependent variable ($p< 0.05$) retained. For both the univariable and multivariable logistic regressions, Odds Ratios will be presented, alongside 95% confidence intervals, and p-values. Given the heterogeneous nature of the study population, subgroup analysis will be performed by stratifying the population by age and sex The same method of modelling will be used for the subgroup analyses for age (15-19 years vs. 20-29 years vs. 30-39 years) and sex (male vs female). The subgroup analysis will be exploratory and with results used to inform subsequent studies.

Qualitative analysis

Audio recordings will be transcribed verbatim. NVivo V.12 software (NVivo qualitative data analysis software, V.12, 2018; QSR International Pty.) will be used to assist data analysis. An inductive content analysis approach [86] will be adopted to analyse transcriptions. Only manifest content will be analysed and the following steps will be undertaken: (1) each interview transcript will be read multiple times; (2) meaning units will be identified; (3) meaning units will be labelled used descriptive codes; (4) codes will be sorted into themes and subthemes.[86] To ensure trustworthiness [87] the following strategies will be adopted: (1) two members of the research team, one researcher with experience of qualitative research and one research assistant trained in qualitative research, will analyse each interview separately with any discrepancies in analysis discussed; (2) discussion of emerging themes with the wider study team; (3) audit trails; (4) triangulation; and (5) disconfirming case analysis.[88] Further, extracts of data supporting each theme and subtheme will be presented to further improve the transparency of the analysis.[89]

Patient and public involvement

The protocol was developed without patient and public involvement (PPI). However, we will seek to involve AYACCS in pilot-testing the survey and in data interpretation of the content analysis of the embedded semi-structured interviews. Specifically, a consultation approach to PPI will be adopted [90] in pilot-testing the survey and a panel of AYACCS research partners will be asked to complete the survey (online and paper based) and comment on the feasibility, acceptability, and relevancy of the survey. Further, results of the content analysis will be presented to the panel to explore whether themes identified by the research team reflect their own experiences. The perspective of the AYACCS research partners will be incorporated into the interpretation of results and their involvement will be reported in accordance with Guidance for Reporting Involvement of Patients and the Public (GRIPP) – short form.[91]

DISCUSSION

To the best of our knowledge, this study will be the first study to examine mental health help-seeking behaviour and attitudes towards e-MH support in an AYACCS population. Results will provide an understanding of: (1) potential associations between predisposing, enabling, environmental, and needs related health service use factors, and help-seeking behaviour; (2) attitudes towards e-MH; and (3) perceived need and preferences for mental health support, alongside barriers and facilitators to help seeking in the population. Our study design allows us to collect both qualitative and quantitative data and therefore will provide a more in-depth and rich understanding of mental health help-seeking behaviour and attitudes towards e-MH support in an AYACCS

For peer review only

population. The identification of factors both positively and negatively associated with help-seeking behaviour may help identify potential targets to improve help-seeking behaviour in the future. For example, interventions could be developed to target and overcome factors found to be negatively associated with help-seeking [92] and subsequently enhance help-seeking behaviours in an AYACCS population in the future. Further, increasing our understanding of attitudes towards e-MH support and preferences regarding the provision of mental health support in the population may inform the development of more acceptable and relevant interventions for this underserved population. In addition, examining preferences for support will enable the development of future psychological interventions that are specifically tailored to and target the needs on an AYACCS population seeking support in relation to their experience of cancer.

Despite the strengths of this study, the design presents some limitations. First, only those AYACCS who report receiving mental health support in the past six months are defined as help-seeking. However, this approach may lead to the exclusion of AYACCS who have attempted to seek formal help but not accessed. Second, the cross-sectional design does not allow us to draw any conclusions regarding cause and effect. Third, the study does not offer any compensation to participants. Research suggests compensation can facilitate the recruitment of minority populations and those from lower socioeconomic backgrounds.[93] Subsequently, this may limit the representativeness of the sample and generalizability of results, especially to marginalized young adults who may already be at an elevated risk of not seeking mental health support.[94] Forth, the current study includes a broad age range (16-39) and it may be expected that adolescents will have differing needs and preferences to young adults in their 20s or 30s. Indeed, a common limitation of research in the area is the inclusion of heterogeneous samples across different age groups.[95] However, the study is designed to be a first step towards developing more acceptable and relevant interventions for the population and may inform future research with specific AYACCS subgroups, taking into account a number of sociodemographic and clinical factors (e.g., gender, current age, age at diagnosis and cancer type). Fifth, the length of the survey may lead to respondent fatigue (e.g., lower levels of attention and motivation in later sections of the questionnaire) potentially resulting in poorer quality data or missing data. Finally, we will not evaluate response bias via comparisons of respondents versus non-responders for mailed surveys.

In conclusion, results of the study may have the potential to improve access to tailored, relevant, and acceptable mental health support for this currently underserved population.

ETHICS AND DISSEMINATION

The study is approved by the Swedish Ethical Review Authority (Dnr: 2020-06271). The rights and welfare of participants will be ensured by all research being conducted in accordance with the Helsinki Declaration. Informed consent will be collected, ensuring participants are aware of requirements for study participation. Participants will be free to withdraw from the study at any time, without providing reason. Contact details for the principal investigator (co-author LvE), the U-CARE Health and Safety Officer, and Uppsala University Data Protection Officer will be provided to all participants should there be any cause for concern regarding the conduct of the study.

For peer review only

All data will be handled according to the Patient Data Act (2008:355) and General Data Protection Regulation (EU 2016/679). Data collected via the online survey via the U-CARE-portal will be securely stored on Uppsala University servers. Data collected via the paper-based survey will be entered into an Access Database, stored on Uppsala University servers. Paper questionnaires will be stored in locked secure filing cabinets, only accessible by authorised members of the study team. Participant contact details for the interview study will be stored separately on a USB stick in a locked secure filing cabinet, separate from study data. Interviews will be audio recorded, with audio files uploaded onto and stored on Uppsala University secure server, using participant identifier numbers and immediately deleted from devices. Interview transcripts will omit any personally identifiable data and will be stored on a secure server.

Survey results will be reported in line with the Checklist for Reporting Results of Internet e-Surveys (CHERRIES) [96] and results of the embedded qualitative interview study will be reported in accordance with the Standards for Reporting Qualitative Research (SRQR).[97] Results will be published in scientific publications in peer reviewed journals and conference presentations. Lay language summaries will also be provided to all community-based organisations and support groups who support recruitment into the study.

AUTHOR'S CONTRIBUTIONS

JW: Conceptualisation, methodology, writing - original draft, supervision, project administration, funding acquisition; JM: Methodology, resources, writing - original draft, project administration; ÅC: Writing – review and editing; PE: Writing – review and editing; AH-S: Writing – review and editing; JH: Resources, writing – review and editing, project administration; MK: Methodology, resources, writing – review and editing, project administration; HPS: Resources, writing – review and editing, project administration; LvE: Methodology, Writing – review and editing, supervision, funding acquisition.

FUNDING

This work is supported by U-CARE, which is a Strategic Research environment funded by the Swedish Research Council (Dnr 2009–1093) and Fredrik O Ingrid Thurings Stiftelse (grant number 2018-00437).

COMPETING INTERESTS

The authors have no competing interests to declare.

COPYRIGHT AND AUTHORS' RIGHTS

I Joanne Woodford, the Submitting Author has the right to grant and does grant on behalf of all authors of the Work (as defined in the below author licence), an exclusive licence and/or a non-exclusive licence for contributions from authors who are: i) UK Crown employees; ii) where BMJ has agreed a CC BY licence shall apply, and/or iii) in accordance with the terms applicable for US Federal Government officers or employees acting as part of their official duties; on a worldwide, perpetual, irrevocable, royalty-free basis to BMJ Publishing Group Ltd ("BMJ") its licensees and where the relevant Journal is co-owned by BMJ to the co-owners of the Journal, to publish the Work in [BMJ Open] and any other BMJ products and to exploit all rights, as set out in our licence.

For peer review only

References

1. Gatta G, Botta L, Rossi S, et al. Childhood cancer survival in Europe 1999-2007: results of EUROCARE-5--a population-based study. *Lancet Oncol* 2014;15:35-47. doi:10.1016/S1470-2045(13)70548-5.
2. Armstrong GT, Kawashima T, Leisenring W, et al. Aging and risk of severe, disabling, life-threatening, and fatal events in the childhood cancer survivor study. *J Clin Oncol* 2014;32:1218-27. doi:10.1200/JCO.2013.51.1055.
3. Bhakta N, Liu Q, Ness KK, et al. The cumulative burden of surviving childhood cancer: an initial report from the St. Jude lifetime cohort study. *Lancet* 2017;390:2569–82. doi:10.1016/S0140-6736(17)31610-0
4. Chow EJ, Ness KK, Armstrong GT, et al. Current and coming challenges in the management of the survivorship population. *Semin Oncol* 2020;47(1):23-39. doi: 10.1053/j.seminoncol.2020.02.007.
5. Robison LL, Hudson MM. Survivors of childhood and adolescent cancer: life-long risks and responsibilities. *Nat Rev Cancer* 2014;14(1):61-70. doi:10.1038/nrc363423.
6. Winick N. Neurocognitive outcome in survivors of pediatric cancer. *Curr Opin Pediatr* 2011; 23(1):27-33. doi: 10.1097/MOP.0b013e32834255e9.
7. Kirchhoff AC, Krull KR, Ness KK, et al. Occupational outcomes of adult childhood cancer survivors: A report from the childhood cancer survivor study. *Cancer* 2011;117(13):3033-3044. doi:10.1002/cncr.25867.
8. Lancashire ER, Frobisher C, Reulen RC et al. Educational attainment among adult survivors of childhood cancer in Great Britain: a population-based cohort study. *J Natl Cancer Inst* 2010;102(4):254-270. doi:10.1093/jnci/djp498.
9. Ander M, Grönqvist H, Cernvall M, et al. Development of health-related quality of life and symptoms of anxiety and depression among persons diagnosed with cancer during adolescence: a 10-year follow-up study. *Psychooncology* 2016;25:582-89. doi:26361085.
10. Brinkman TM, Zhu L, Zeltzer LK, et al. Longitudinal patterns of psychological distress in adult survivors of childhood cancer. *Br J Cancer* 2013;109:1373-81. doi:10.1038/bjc.2013.428.
11. Seitz DC, Besier T, Debatin KM, et al. Posttraumatic stress, depression and anxiety among adult long-term survivors of cancer in adolescence. *Eur J Cancer* 2010;46:1596-06. doi:10.1016/j.ejca.2010.03.001.
12. Patton GC, Sawyer SM, Santelli JS, et al. Our future: a lancet commission on adolescent health and wellbeing. *Lancet* 2016;387:2423-78. doi:10.1016/S0140-6736(16)00579-1.
13. Blackmore SJ. Adolescence and mental health. *Lancet* 2019;393:2030-31. doi:10.1016/S0140-6736(19)31013-X.
14. Ahomäki R, Kero A, Koivisto M, et al. Purchases of antidepressants after cancer at a young age in Finland. *Int J Cancer* 2019:144:1227-33. doi:10.1002/ijc.31942 25.
15. Johannsdottir IM, Loge JH, Kiserud CE, et al. Increased prescription rates of anxiolytics and hypnotics to survivors of cancer in childhood, adolescence, and young adulthood-a population-based study. *Pediatr Blood Cancer* 2018;65:e26860. doi:10.1002/pbc.26860.
16. Brinkman TM, Ullrich NJ, Zhang N, et al. Prevalence and predictors of prescription psychoactive medication use in adult survivors of childhood cancer: a report from the childhood cancer survivor study. *J Cancer Surviv* 2013;7:104–14. doi:10.1007/s11764-012-0250-x.

For peer review only

17. Gunnes MW, Lie RT, Bjørge T, et al. Suicide and violent deaths in survivors of cancer in childhood, adolescence and young adulthood-a national cohort study. *Int J Cancer* 2017;140:575-80. doi: 10.1002/ijc.30474.

18. Bleyer A, Barr R, Ries L, etal. *Cancer in adolescents and young adults.* Springer International Publishing, 2017.

19. Bibby H, White V, Thompson K, et al. What are the unmet needs and care experiences of adolescents and young adults with cancer? A systematic review. *J Adolesc Young Adult Oncol* 2017;6:6-30. doi:10.1089/jayao.2016.0012.

20. Kaul S, Avila JC, Mutambudzi M, et al. Mental distress and health care use among survivors of adolescent and young adult cancer: a cross-sectional analysis of the national health interview survey. *Cancer* 2017;123:869-78. doi:10.1002/cncr.30417.

21. Olsson M, Jarfelt M, Pergert P, et al. Experiences of teenagers and young adults treated for cancer in Sweden. *Eur J Pncol Nurs* 2015;19:575-81. doi:10.1016/j.ejon.2015.03.003.

22. Svedberg P, Einberg EL, Wärnestål P, et al. Support from healthcare services during transition to adulthood – experiences of young adult survivors of pediatric cancer. *Eur J Oncol Nursing* 2016;21:105-12. doi:10.1016/j.ejon.2016.02.008.

23. Rebello TJ, Marques A, Gureje O, et al. Innovative strategies for closing the mental health treatment gap globally. *Curr Opin Psychiatry* 2014;27:308-14. doi:10.1097/YCO.0000000000000068.

24. Kim MA, Yi J. Life after cancer: how does public stigma increase psychological distress of childhood cancer survivors? *Int J Nurs Stud* 2014;51:1605-14. doi:10.1016/j.ijnurstu.2014.04.005.

25. Riper H, Andersson G, Christensen H, et al. Theme issue on e-mental health: a growing field in internet research. *J Med Internet Res* 2010;12:e74. doi:10.2196/jmir.1713.

26. Patel V, Saxena S, Lund C, et al. The lancet commission on global mental health and sustainable development. *Lancet* 2018;392:1553-98. doi:10.1016/S0140-6736(18)31612-X.

27. Andersson G. Internet interventions: past, present and future. *Internet Interv* 2018;12:181- 88. doi: 10.1016/j.invent.2018.03.008.

28. Bunge EL, Dickter B, Jones MK, et al. Behavioural intervention technologies and psychotherapy with youth: a review of the literature. *Curr Psychiatry Rev* 2016;12:14–28. doi:10.2174/1573400511666150930232254

29. Carlbring P, Andersson G, Cuijpers P, et al. Internet-based vs. face-to-face cognitive behaviour therapy for psychiatric and somatic disorders: an updated systematic review and meta-analysis. *Cogn Behav Ther* 2018;47:1-18. doi:10.1080/16506073.2017.1401115.

30. Sansom-Daly UM, Wakefield CE, Bryant RA, et al. Online group-based cognitive-behavioural therapy for adolescents and young adults after cancer treatment: a multicenter randomised controlled trial of recapture life-AYA. *BMC Cancer* 2012;12. doi:10.1186/1471-2407-12-339.

31. Hagström, J, Ander, M, Cernvall, et al. Heeding the psychological concerns of young cancer survivors: a single-arm feasibility trial of CBT and a cognitive behavioural conceptualization of distress. *PeerJ* 2020;8:e8714. doi:10.7717/peerj.8714.

For peer review only

32. Younes N, Chollet A, Menard E, et al. E-mental health care among young adults and help-seeking behaviours: a transversal study in a community sample. *J Med Internet Res* 2015;17:e123. doi: 10.2196/jmir.4254.

33. Ander M, Wikman A, Ljótsson B, et al. Guided internet-administered self-help to reduce symptoms of anxiety and depression among adolescents and young adults diagnosed with cancer during adolescence (U-CARE: YoungCan): a study protocol for a feasibility trial. BMJ Open 2017;7: e013906. doi:10.1136/bmjopen-2016-013906.

34. Ander M. *Cancer during adolescence - psychological consequences and development of psychological treatment.* 2017. http://uu.diva-portal.org/smash/record.jsf?pid=diva2%3A1153609&dswid=1594

35. Barnett M, McDonnell G, DeRosa A, et al. Psychosocial outcomes and interventions among cancer survivors diagnosed during adolescence and young adulthood (AYA): a systematic review. *J Cancer Surviv* 2016;10:814-31. doi:10.1007/s11764-016-0527-6.

36. Devine KA, Viola AS, Coups EJ, et al. Digital health interventions for adolescent and young adult cancer survivors. *JCO Clin Cancer Inform* 2018;2:1-15. doi:10.1200/CCI.17.00138.

37. Moody L, Turner A, Osmond J, et al. Web-based self-management for young cancer survivors: consideration of user requirements and barriers to implementation. *J Cancer Surviv* 2015;9:188–200. doi:10.1007/s11764-014-0400-4.

38. Seitz DC, Knaevelsrud C, Duran G, et al. Efficacy of an internet-based cognitive-behavioral intervention for long-term survivors of pediatric cancer: a pilot study. *Support Care Cancer* 2014;22:2075–83. doi:10.1007/s00520-014-2193-4.

39. van Ballegooijen W, Cuijpers P, van Straten A, et al. Adherence to internet-based and face-to-face cognitive behavioural therapy for depression: a meta-analysis. *PLoS One* 2014;9:e100674. doi:10.1371/journal.pone.0100674.

40. Nilsson A, Sörman K, Klingvall J, et al. MyCompass in a Swedish context - lessons learned from the transfer of a self-guided intervention targeting mental health problems. *BMC Psychiatry* 2019;19:51. doi:10.1186/s12888-019-2039-1.

41. Woodford J, Farrand P, Bessant M, et al. Recruitment into a guided internet based CBT (iCBT) intervention for depression: lesson learnt from the failure of a prevalence recruitment strategy. *Contemp Clin Trials* 2011;32:641-48. doi:10.1016/j.cct.2011.04.013.

42. Rapp AM, Chavira DA, Sugar CA, et al. Integrated primary medical-behavioural health care for adolescent and young adult depression: predictors of service use in the youth partners in care trial. *J Pediatr Psychol* 2017;42:1051-64. doi:10.1093/jpepsy/jsx057.

43. Gulliver A, Griffiths KM, Christensen H. Perceived barriers and facilitators to mental health help-seeking in young people: a systematic review. *BMC Psychiatry* 2010;10. doi:10.1186/1471-244X-10-113.

44. Kaess M, Bauer S. Promoting help-seeking using e-technology for adolescents: the ProHEAD consortium. *Trials* 2019;20:72. doi:10.1186/s13063-018-3162-x.

45. Lehmann V, Grönqvist H, Engvall G, et al. Negative and positive consequences of adolescent cancer 10 years after diagnosis: an interview-based longitudinal study in Sweden. *Psychooncology* 2014;23:1229-35. doi:10.1002/pon.3549.

For peer review only

46. Ander M, Thorsell Cederberg J, von Essen L, et al. Exploration of psychological distress experienced by survivors of adolescent cancer reporting a need for psychological support. *PLoS One* 2018;13:e0195899. doi:10.1371/journal.pone.0195899.

47. Wilford J, Buchbinder D, Fortier MA, et al. "She was a little social butterfly": a qualitative analysis of parent perception of social functioning in adolescent and young adult brain tumor survivors. *J Pediatr Oncol Nurs* 2017;34:239-49. doi:10.1177/1043454216688660.

48. Andersen RM. Revisiting the behavioral model and access to medical care: does it matter? *J Health Soc Behav* 1995;36:1-10. doi:10.2307/2137284

49. Bock JO, Hajek A, König HH. The longitudinal association between psychological factors and health care use. *Health Serv Res* 2018;53:1065-91. doi:10.1111/1475-6773.12679.

50. Gudleski GD, Satchidanand N, Dunlap LJ, et al. Predictors of medical and mental health care use in patients with irritable bowel syndrome in the United States. *Behav Res Ther* 2017;88:65-75. doi:10.1016/j.brat.2016.07.006.

51. Mills V, Van HM, Baur J, et al. Predictors of mental health service utilisation in a non-treatment seeking epidemiological sample of Australian adults. *Community Ment Health J* 2012;48:511-21. doi:10.1007/s10597-011-9439-0.

52. Mooney-Somers J, Lewis P, Kerridge I. Discursive constructions of youth cancer: findings from creative methods research with healthy young people. *J Cancer Surviv* 2016;10:427-36. doi:10.1007/s11764-015-0488-1.

53. van Schoors M, Caes L, Knoble NB, et al. Systematic review: associations between family functioning and child adjustment after pediatric cancer diagnosis: a meta-analysis. *J Pediatr Psychol* 2017;42:6-18. doi:10.1093/jpepsy/jsw070.

54. Sweeney GM, Donovan CL, March S, et al. Logging into therapy: adolescent perceptions of online therapies for mental health problems. *Internet interv* 2016;15:93-9. doi:10.1016/j.invent.2016.12.001.

55. Wetterlin FM, Mar MY, Neilson EK, et al. eMental health experiences and expectations: a survey of youths' web-based resource preferences in Canada. *J Med Internet Res* 2014;16:e29. doi:10.2196/jmir.3526.

56. Hollis C, Morris R, Martin J, et al. Technological innovations in mental healthcare: harnessing the digital revolution. *Br J Psychiatry* 2015;206:263-265. doi:10.1192/bjp.bp.113.142612.

57. Fleming T, Bavin L, Lucassen M, et al. Beyond the trial: systematic review of real-world uptake and engagement with digital self-help interventions for depression, low mood, or anxiety. *J Med Internet Res* 2018;20:e199. dio:10.2196/jmir.9275.

58. Apolinário-Hagen J, Kemper J, Stürmer C. Public acceptability of e-mental health treatment services for psychological problems: a scoping review. *JMIR Ment Health* 2017;4:e10. doi:10.2196/mental.6186.

59. van Beugen S, Ferwerda M, Hoeve D, et al. Internet-based Ccgnitive behavioral therapy for patients with chronic somatic conditions: a meta-analytic review. *J Med Internet Res* 2014;16(3):e88. doi: 10.2196/jmir.2777.

60. Radomski AD, Wozney L, McGrath P, et al. Design and delivery features that may improve the use of internet-based cognitive behavioral therapy for children and adolescents with anxiety: a realist literature

For peer review only

synthesis with a persuasive systems design perspective. J Med Internet Res 2019;21(2):e11128. doi: 10.2196/11128.

61. Ferwerda M, van Beugen S, van Burik A, et al. What patients think about e-health: patients' perspective on internet-based cognitive behavioral treatment for patients with rheumatoid arthritis and psoriasis. *Clin Rheumatol* 2013;32(6):869-873. doi:10.1007/s10067-013-2175-9.

62. Barakat LP, Galtieri LR, Szalda D, Schwartz LA. Assessing the psychosocial needs and program preferences of adolescents and young adults with cancer. *Support Care Cancer 2016*;24(2):823-832. doi:10.1007/s00520-015-2849-8.

63. Grönqvist H, Olsson EMG, Johansson B, et al. Fifteen challenges in establishing a multidisciplinary research program on eHealth research in university settings: a case study. *J Med Internet Res* 2017;19:e173. doi:10.2196/jmir.7310.

64. Kilsdonk E, van Dilmen-den Broeder E, van der Pal HJ, et al. Effect of web-based versus paper-based questionnaires and follow-up strategies on participation rates of Dutch childhood cancer survivors: a randomized controlled trial. *JMIR Cancer* 2015;1:e11. doi:10.2196/cancer.3905.

65. Brick JM, Williams D. Explaining rising nonresponse rates in cross-sectional surveys. *AAPSS* 2013;645:36–59. doi:10.1177/0002716212456834.

66. Batterham PJ. Recruitment of mental health survey participants using internet advertising: content, characteristics and cost effectiveness. *Int J Methods Psychiatr Res* 2014;23:184-91. doi: 10.1002/mpr.1421.

67. Gorman JR, Roberts SC, Dominick, SA. A Diversified recruitment approach incorporating social media leads to research participation among young adult-aged female cancer survivors. *J Adolesc Young Adult Oncol* 2014:3: 59–5. doi:10.1089/jayao.2013.0031.

68. O'Connor A, Jackson L, Goldsmith L, et al. Can I get a retweet please? Health research recruitment and the twittersphere. *J Adv Nurs* 2014;70:599-09. doi:10.1111/jan.12222.

69. Kilsdonk E, Wendel E, van Dulmen-den Broeder E, et al. Participation rates of childhood cancer survivors to self-administered questionnaires: a systematic review. *Eur J Cancer Care* 2017;26:e12462. doi:10.1111/ecc.12462.

70. Boerema AM, Kleiboer A, Beekman ATet al. Determinants of help-seeking behavior in depression: a cross-sectional study. *BMC Psychiatry* 2016;16. doi:10.1186/s12888-016-0790-0.

71. Apolinário-Hagen J, Vehreschild V, Alkoudmani RM. Current views and perspectives on e-mental health: an exploratory survey study for understanding public attitudes toward internet-based psychotherapy in Germany. *JMIR Ment Health* 2017;4:e8. doi:10.2196/mental.6375.

72. Apolinário-Hagen J, Harrer M, Kählke F, et al. Public attitudes toward guided internet-based therapies: web-based survey study. *JMIR Ment Health* 2018;5:e10735. doi:10.2196/10735.

73. Alfonsson S, Wallin E, Maathz P. Factor structure and validity of the depression, anxiety and stress scale-21 in Swedish translation. *J Psychiatr Ment Health Nurs* 2017;24:154-62. doi:10.1111/jpm.

74. Brown TA, Chorpita BF, Korotitsch W, et al. Psychometric properties of the depression anxiety stress scales (DASS) in clinical samples. *Behav Res Ther* 1997;35:79-89. doi:10.1016/s0005-7967(96)00068-x.

For peer review only

75. Ng F, Trauer T, Dodd S, et al. The validity of the 21-item version of the depression anxiety stress scales as a routine clinical outcome measure. *Acta Neuropsychiatr* 2007;19:304-10. doi:10.1111/j.1601-5215.2007.00217.x.
76. Sinclair SJ, Siefert CJ, Slavin-Mulford JM, et al. Psychometric evaluation and normative data for the depression, anxiety, and stress scales-21 (DASS-21) in a nonclinical sample of U.S. adults. *Eval Health Prof* 2012;35:259-79. doi:10.1177/0163278711424282.
77. Bottesi G, Ghisi M, Altoè G, etal. The Italian version of the depression anxiety stress scales-21: factor structure and psychometric properties on community and clinical samples. *Compr Psychiatry* 2015;60:170-81. doi: 10.1016/j.comppsych.2015.04.005.
78. Komiya N, Good GE, Sherrod NB. Emotional openness as a predictor of college students' attitudes toward seeking psychological help. *J Couns Psychol* 2000;47:138–43. doi:10.1037/AJ022-0167,47.1.138
79. Bathje GJ, Pryor JB. The relationships of public and self-stigma to seeking mental health services. *J Ment Health Couns* 2011;33:161–76. doi:10.17744/mehc.33.2.g632039274160411.
80. Pinto MD, Greenblatt AM, Hickman RL, et al. Assessing the critical parameters of eSMART-MH: a promising avatar-based digital therapeutic intervention to reduce depressive symptoms. *Perspect Psychiatr Care* 2016;52:157-68. doi:10.1111/ppc.12112.
81. Cohen S, Mermelstein R, Kamarck T, et al. Measuring the functional components of social support. In: Sarason IG, Sarason BR, eds. *Social support: theory, research, and applications*. Martinus Nijhoff 1985. 73–94.
82. Jung H, von Stenberg K, Davis K. Expanding a measure of mental health literacy: development and validation of multicomponent mental health literacy measure. *Psychiatry Res* 2016;243:278-86. doi:10.1016/j.addr.2016.07.002.
83. Fusch PI, Ness LR. Are we there yet? Data saturation in qualitative research. *Qual Rep* 2015;20:1408-1416.
84. Guest G, Bunce A, Johnson L. How many interviews are enough? An experiment with data saturation and variability. *Sage journal* 2006;18:59-82. doi:10.1177/1525822X05279903.
85. Sim J., Saunders B, Waterfield J, Kingstone T. Can sample size in qualitative research be determined a priori? *Int J Soc Res Methodol* 2018; 21:5, 619-634. doi: 10.1080/13645579.2018.1454643.
86. Elo S, Kyngäs H. The qualitative content analysis process. *J Adv Nurs*. 2008;62:107-15. doi:10.1111/j.1365-2648.2007.04569.x.
87. Krefting L. Rigor in qualitative research: the assessment of trustworthiness. *Am J Occup Ther* 1991;45:214-22. doi:10.5014/ajot.45.3.214 [published Online First: 1 March 1991].
88. Patton MQ. Enhancing the quality and credibility of qualitative analysis. *Health Serv Res* 1999;34:1189–1208.
89. Elliott R, Fischer CT, Rennie DL. Evolving guidelines for publication of qualitative research studies in psychology and related fields. *Br J Clin Psychol*. 1999;38:215-29. doi:10.1348/014466599162782.
90. Jennings H., Slade M., Bates P. et al. Best practice framework for patient and public involvement (PPI) in collaborative data analysis of qualitative mental health research: methodology development and refinement. *BMC Psychiatry*. 2018:18. doi:10.1186/s12888-018-1794-8.

For peer review only

91. Staniszewska S, Brett J, Simera I, et al. GRIPP2 reporting checklists: tools to improve reporting of patient and public involvement in research. *BMJ* 2017;358:j3453. doi:10.1136/bmj.j3453.

92. Kauer SD, Mangan C, Sanci L. Do online mental health services improve help-seeking for young people? A systematic review. *J Med Internet Res* 2014;16:e66. doi:10.2196/jmir.3103.

93. Kost RG, de Rosa JC. Impact of survey length and compensation on validity, reliability, and sample characteristics for Ultrashort-, Short-, and Long-Research Participant Perception Surveys. *J Clin Transl Sci*. 2018;2(1):31-37. doi:10.1017/cts.2018.18.

94. Sapiro, B., Ward, A. Marginalized youth, mental health, and connection with others: a review of the literature. *Child Adolesc Soc Work* 2020; J37:343–357. doi.10.1007/s10560-019-00628-5.

95. Warner EL, Kent EE, Trevino KM, et al. Social well-being among adolescents and young adults with cancer: a systematic review. *Cancer* 2016;122(7):1029-1037. doi:10.1002/cncr.29866.

96. Eysenbach G. Improving the quality of web surveys: the checklist for reporting results of internet e-surveys (CHERRIES). J Med Internet Res 2004;6:e34. doi:10.2196%2Fjmir.6.3.e34.

97. O'Brien BC, Harris IB, Beckman TJ, et al. Standards for reporting qualitative research: a synthesis of recommendations. *Academic Medicine* 2014;89:1245-51. doi:10.1097/ACM.0000000000000388.

For peer review only
